# The Effect of Diet and Exercise on Intestinal Integrity and Microbial Diversity in Mice

**DOI:** 10.1371/journal.pone.0150502

**Published:** 2016-03-08

**Authors:** Sara C. Campbell, Paul J. Wisniewski, Michael Noji, Lora R. McGuinness, Max M. Häggblom, Stanley A. Lightfoot, Laurie B. Joseph, Lee J. Kerkhof

**Affiliations:** 1 Department of Exercise Science and Sports Studies, Rutgers University, New Brunswick, NJ 08901, United States of America; 2 Department of Marine and Coastal Sciences, Rutgers University, New Brunswick, NJ 08901, United States of America; 3 Department of Biochemistry and Microbiology, Rutgers University, New Brunswick, NJ 08901, United States of America; 4 Chief Pathologist, Oklahoma City, OK, United States of America; 5 Department of Pharmacology and Toxicology, Rutgers University, New Brunswick, NJ 08901, United States of America; National Institute of Agronomic Research, FRANCE

## Abstract

**Background:**

The gut microbiota is now known to play an important role contributing to inflammatory-based chronic diseases. This study examined intestinal integrity/inflammation and the gut microbial communities in sedentary and exercising mice presented with a normal or high-fat diet.

**Methods:**

Thirty-six, 6-week old C57BL/6NTac male mice were fed a normal or high-fat diet for 12-weeks and randomly assigned to exercise or sedentary groups. After 12 weeks animals were sacrificed and duodenum/ileum tissues were fixed for immunohistochemistry for occludin, E-cadherin, and cyclooxygenase-2 (COX-2). The bacterial communities were assayed in fecal samples using terminal restriction fragment length polymorphism (TRFLP) analysis and pyrosequencing of 16S rRNA gene amplicons.

**Results:**

Lean sedentary (LS) mice presented normal histologic villi while obese sedentary (OS) mice had similar villi height with more than twice the width of the LS animals. Both lean (LX) and obese exercise (OX) mice duodenum and ileum were histologically normal. COX-2 expression was the greatest in the OS group, followed by LS, LX and OX. The TRFLP and pyrosequencing indicated that members of the *Clostridiales* order were predominant in all diet groups. Specific phylotypes were observed with exercise, including *Faecalibacterium prausnitzi*, *Clostridium* spp., and *Allobaculum* spp.

**Conclusion:**

These data suggest that exercise has a strong influence on gut integrity and host microbiome which points to the necessity for more mechanistic studies of the interactions between specific bacteria in the gut and its host.

## Introduction

Microbiome dysbiosis has proven to be a major contributor to chronic gut inflammatory diseases, like Irritable Bowel Syndrome (IBS) and ulcerative colitis [1]. Interestingly, exercise has been observed to improve the quality of life for IBS patients, but generally the influence of exercise on intestinal health is poorly understood [2,3]. Other groups have explored the link between diet and dysbiosis but there is limited evidence on the combined interaction of gut microbiome, diet and exercise [4,5]. This work has sought to explore the symbiotic relationship between host behavior, through diet and exercise, by investigating the histopathological alterations in the gut through specific biomarkers of inflammation, gut integrity and gut microbial ecology.

The human and animal gut microbiome has received widespread attention due its role in energy harvesting and contributing to chronic systemic low grade inflammation [6–8]. The delicate balance between members of the phyla *Firmicutes* and *Bacteroidetes* are important in determining the metabolic phenotype of the host [6, 9–11], This has been shown in animal studies where high-fat diets alter the relative abundance of *Firmicutes* and *Bacteroidetes* [6,8, 11–13]. Recent studies suggest that the delicate balance between key opportunistic pathogens, e.g. Enterobacter spp. [14] and favorable bacteria, e.g. *Akkermansia muciniphilia* [5,15] is especially critical for homeostasis.

Obesity has risen dramatically throughout the world and is often associated with inflammation, comorbidities and disability [16,17]. The term “leaky gut” was coined by Gummesson, who related lower gastrointestinal (GI) leakiness and adiposity, which in turn produces low grade systemic inflammation resulting from metabolic endotoxemia [18], while Cani observed that leaky gut can also be associated with diabetes and obesity [12]. Leaky gut results from dysbiosis in the gut microbiota. Dysbiois promotes the release of endotoxin and flagellin from gram negative bacteria, which bind to TLR5, inducing inflammation and damage of the intestinal epithelium [12,19]. Interestingly, altered intestinal integrity has also been associated with high-fat diets and breakdown of tight junction proteins, occludin and zona-occludin-1 (ZO-1) [12,20–22]. Regulation of this barrier by tight junction proteins is dynamic representing a balance between the pore pathway and leak pathway. The pore pathway regulates the permeability of the tight junction to ions and small molecules, whereas the leak pathway regulates the permeability of macromolecules [23]. The pore pathway appears to be dependent upon the cadherins and claudins while the leak pathway is dependent upon the ZO-1 and the occludins [24,25].

Obesity is associated with many pathological conditions and higher levels of inflammatory cytokines. Interlukin-6 (IL-6) is one of the most prominent inflammatory cytokines in obesity and diabetes research because its serum concentration positively correlates with increased fat mass [26]. The secretion IL-6 is complex as evidence suggests that this cytokine can have both detrimental effects in obesity and yet positive effects on tissue homeostasis and potential to resolve inflammation [27]. The totality of evidence suggests that inflammation and dysmetabolism seem to be more a consequence rather than a cause of obesity [28].

Exercise is often prescribed for weight loss and maintenance. Evidence suggests that chronic exercise typically causes a partial but incomplete compensation in energy intake and this is likely due to beneficial changes in appetite-regulating hormones [29]. Notably increases in peptide YY (PYY) and decreases in ghrelin have been reported [30–33]. PYY mediates its effects via G-protein coupled receptors and primarily acts in the brain to inhibit food intake [34]. Additionally, it has been shown to increase energy expenditure and fat oxidation rates [35]. Ghrelin levels are higher prior to meals and lower after meals, suggesting it may have a role in weight gain and meal initiation. Since ghrelin is still considered one of the only circulating appetite stimulants, the ability of exercise to reduce this hormone may promote weight control by reducing ones desire to consume meals [29,36].

It has also been observed that exercise induces a diverse microbiome [5]. This is an important observation, though exercise has not been thoroughly linked to gut integrity. Existing evidence in this area suggests that intense, prolonged exercise, in the heat, will cause leaky gut in endurance athletes [37]. This is an interesting observation since exercise normally reduces the risk of GI cancer, reflux, and incidence of ulcers, fatty liver, IBS and diverticulitis [38]. In addition, exercise in older animals was shown to reduce expression of inflammatory mediators and apoptotic markers in intestinal lymphocytes, suggesting a protective role for exercise in intestinal health [39,40]. Furthermore, exercise may enhance butyrate producing cecal bacteria, which are known to reduce inflammation and promote cell proliferation [41]. Exercise may protect the morphology and integrity of the intestine, and reduce systemic inflammation, even in the presence of a high fat diet.

Therefore, the purpose of this study was to examine intestinal integrity and gut microbial ecology in sedentary and exercised animals on normal and high-fat diets. Our hypothesis was two-fold: (1) exercise reduces intestinal inflammation in the high-fat fed animals; and (2) exercise promotes an anti-obesogenic microbiome.

## Methods

### Animals, Diets and Exercise

All animals received humane care in compliance with the institution's guidelines, as outlined in the Guide for the Care and Use of Laboratory Animals published by the National Institutes of Health. Experiments were completed at Rutgers University and approved by the Rutgers University Institutional Animal Care and Use Committee (IACUC). C57BL/6NTac male 6-week old mice (Taconic Farms, Germantown, NY) were housed 1/cage in an environmentally controlled room with a 12 hour light/dark cycle and maintained on the specified diet and water *ad libitum*. The animals were acclimated for two-weeks and upon acclimation randomly assigned to one of four groups: (1) lean sedentary (LS); (2) diet-induced obesity (DIO) sedentary (OS); (3) lean exercise (LX); and (4) DIO exercise (OX) groups. Animals in the lean groups consumed a normal diet (D12450H, 10% kcal from fat, Research Diets, New Brunswick, NJ); and in the DIO groups a high-fat diet (D12451, 45% kcal from fat, Research Diets, New Brunswick, NJ). Food intake was monitored every other day, animals weighed once per week and body composition was determined prior to sacrifice using Echo MRI (Houston, TX). Animals in the exercise groups had access to a free running wheel (Coulbourn Instruments, Allentown, PA) linked to ClockLab (Actimetrics, Willmette, IL) to determine exercise volume.

At the end of 12 weeks animals were sacrificed by live decapitation. All animals in the study were healthy for the duration of the study; however humane endpoints were in place, per the Rutgers University IACUC, if an animal would have become severely ill/moribund prior to the experimental endpoint. Fecal pellets were collected from the distal colon of the mice, snap frozen and stored at -80°C for later analysis. Blood was collected in EDTA coated tubes and centrifuged at 4°C to obtain plasma which was stored at -80°C for later analysis. Tissue analysis was as described below.

### Histochemistry and Immunohistochemistry

#### Histochemistry

Duodenal and ileal intestinal segments were harvested and denoted as follows: the first 4–6 cm from the pyloric sphincter was collected as duodenum and the 4–6 cm above the ileocecal junction was collected as ileum. Tissue was fixed overnight at 4°C in 3% paraformaldehyde and 2% sucrose then embedded in paraffin. For histological analysis, 5 μm intestinal tissue sections were stained with hematoxylin and eosin (H & E) (Goode Histolabs, New Brunswick, NJ). For histomorphometry all tissues sections were blindly scored by a board certified pathologist (Dr. Stanley Lightfoot).

#### Immunohistochemistry

For immunohistochemical studies, 5 μm intestinal sections were deparaffinized, rehydrated and subsequently blocked with 10%, 25% or 100% normal goat serum at room temperature for 2 hr. The tissue sections were then incubated overnight at 4°C with primary rabbit affinity purified polyclonal antibodies against structural components of the intestine as described below, or with rabbit IgG (ProSci Inc., Poway, CA) as a control. Duodenal segments were stained for cyclooxygenase-2 (COX-2, 1:200, Abcam, Cambridge, MA) expression. Ileal segments were stained for COX-2 (1:200, Abcam), occludin (1:50, Thermo Fisher Scientific, Waltham, MA), and E-cadherin (1:300, Abcam). Tissue sections were then incubated for 30 min with a biotinylated goat anti-rabbit secondary antibody (Vector Labs, Burlingame, CA). Antibody binding was visualized using a DAB Peroxidase Substrate Kit (Vector Labs). Tissue sections were photographed using the VS120-S5 System (Olympus, Center Valley, PA). For histomorphometry all tissues sections were blindly scored by a board certified pathologist (Dr. Stanley Lightfoot). Slide staining was reported using a dual number system (#X#). The first number was the intensity of the stain and the second number was the amount of stain present in the specimen. The intensity was graded on 1–3 and the amount indicated as follows- 1-<10%; 2-11-40%; 3-41-60%; and 4->60%. The total score was obtained by multiplying the two numbers.

### Gut Microbial Community Analysis

#### DNA purification, 16S rRNA gene profiling, and pyrosequence analysis of fecal bacteria

The gut bacteria were assayed by terminal restriction fragment length polymorphism (TRFLP) in a single stool sample from triplicate animals in the experimental treatments [42], followed by pyrosequence analysis of 16S rRNA genes of pooled DNA samples per experimental treatment. Genomic DNA was extracted by phenol/chloroform methodology [43]. Small subunit rRNA genes were amplified using 10 ng of genomic DNA, the forward primer 27F ((5’-AGAGTTTGATCCTGGCTCAG-3’; labeled with the fluorochrome 6’-carboxyfluorescein) and the bacterial-specific primer 1100R (5’-AGGGTTGCGCTCGTTG-3’) for TRFLP analysis [43]. Fifteen ng of amplicon was digested with *Mnl-I* endonuclease (New England Biolabs, Beverly, MA) for 6 h and sized using an ABI 310 genetic analyzer (Applied Biosystems, Foster City, CA) with Genescan^™^ software and an internal size standard. Peak detection was set at 25 arbitrary fluorescent units and only peaks representing >0.5% of the overall community profile area were considered for further analysis. Cluster analysis of the profiles was performed by conversions of presence/absence data to A/T and FastTree methods with bootstrap support using Geneious^™^ phylogenetic software, similar to Clark and Warwick [44]. Sorting of the terminal restriction fragment (TRF) areas for heat mapping was initially done in ascending order by counting peaks present in all experimental treatments (LS, OS, LX, OX) and then sub-sorting per treatment in descending order of peak area for ease of comparison. Color coding of the data was in increments of 1000–5000 arbitrary fluorescent units.

Clone libraries from pooled fecal DNA samples from each treatment group were established using gel purified 16S rRNA gene amplicons (GeneClean^®^ II kit; MP Biomedicals Ca. No. 111001400), and the pGEM^®^-T Easy Vector System I kit (Promega Ca. No. A1360). Clones matching specific peaks were identified by TRFLP and sequenced using Sanger methods (GeneWiz, Plainfield, NJ) and are available in Genbank (Ascension numbers: KU644595-KU644645). Phylogenetic analysis was performed using likelihood methods for 379 unambiguously aligned positions with the Geneious^™^ software package.

Pooled fecal DNA samples from each treatment group were also processed for pyrosequencing and community analysis. 16S rRNA genes of the bacterial community were amplified with 27F and 519R primers, then sequenced and analyzed by Molecular Research LP (Shallowater, TX) using a Roche 454 Genome Sequencer following the manufacturer’s guidelines. The sequence data was processed using a proprietary analysis pipeline (MR DNA, Molecular Research LP). Briefly, the barcodes, primers, short sequences (< 200bp) and the sequences containing ambiguous base calls and homopolymer runs (>6bp) were removed to ensure the quality of reads. The remaining sequences were denoised and then chimeras and singleton sequences were removed. Operational taxonomic units (OTUs) were clustered using a sequence similarity threshold of 97% and taxonomically classified by BLASTN against a curated GreenGenes database as described previously [45,46]. These sequences are presented in S1 Table and all 454 sequences are available in Genbank (BioProject# PRJNA 309613).

### Plasma Blood Samples

Plasma samples were screened for metabolic markers using a mouse metabolic hormone kit (Millipore, MMHMAG-44K) on a Luminex MagPix; specific markers analyzed included leptin, ghrelin, insulin, IL-6, C-peptide 2, PYY and pancreatic polypeptide (PP).

### Statistical analysis

Anthropometric data and blood markers were analyzed using one-way ANOVA and Tukey post-hoc test (SPSS 23, IBM SPSS Statistics, Armonk, NY). A difference of mean with a p value of ≤ 0.05 was considered statistically significant.

## Results

### Animals, Body Weights and Exercise

For the exercise groups, LX and OX, wheel counts were not significantly different from each other (Table 1). Animals in the LX group consumed significantly more food per day than the other groups. Furthermore, animals in the OX group consumed significantly more that the sedentary groups which equated to the highest caloric intake of any of the groups. Despite this higher kcal consumption OX animals had lower percent body fat, less total fat mass and reduced body weights compared to their sedentary counterparts (OS) (Table 1). There were no significant differences between the groups with regards to lean mass.

**Table 1:** Animal Characteristics.

	Lean-Sedentary	Lean-Exercise	DIO-Sedentary	DIO-Exercise
Body weight (g)	30.4±3.2^a^	29.7±5.5^a^	42.7±3.2^b^	35.3±7.4^c^
Food Intake (g/day)	3.3±0.3^a^	4.2±0.4^a^	3.3±0.4^b^	3.7±0.5^c^
Body Fat (%)	22.9±6.0^a^	15.3±8.9^b^	42.4±2.5^c^	32.8±11.7^d^
Lean Mass (g)	21.5±1.1	22.8±1.6	22.8±1.2	22.4±2.0
Fat Mass (g)	7.1±2.5^a^	4.9±4.2^a^	18.1±2.1^b^	12.2±5.9^c^
Kcal/day	12.3±1.0^a^	16.0±1.4^b^	15.0±1.7^b^	17.0±2.1^b^
Exercise volume (counts/day)	0^a^	17,390±6,890^b^	0^a^	16,480±6,930^b^

Values are mean ± SD, n = 9 in each group.

Values that do not share the same superscript letters are significantly (*p<0*.*05*) different from each other.

Animals in the LX group had the lowest body weights followed by LS, although these were not significantly different from each other. The OS group had significantly higher body weights compared to the lean groups. It is important to note that the body weights were variable in the OX group, as were the wheel counts per day. Our results indicated that some of the mice consumed on average the same as their exercising counterparts. This was despite a lower exercise volume only ~6,000 counts per day, significantly less than the group average. We also noted that 4 of our 9 OX mice were very lean, averaging 29 grams, (similar to the LX group) and exercised substantially. This observation, anecdotally, appears to be similar to a human population where some may consume a higher fat diet and with exercise can maintain a healthier weight, while others do not.

### High-Fat Diets Alter Intestinal Morphology

**Fig 1:**
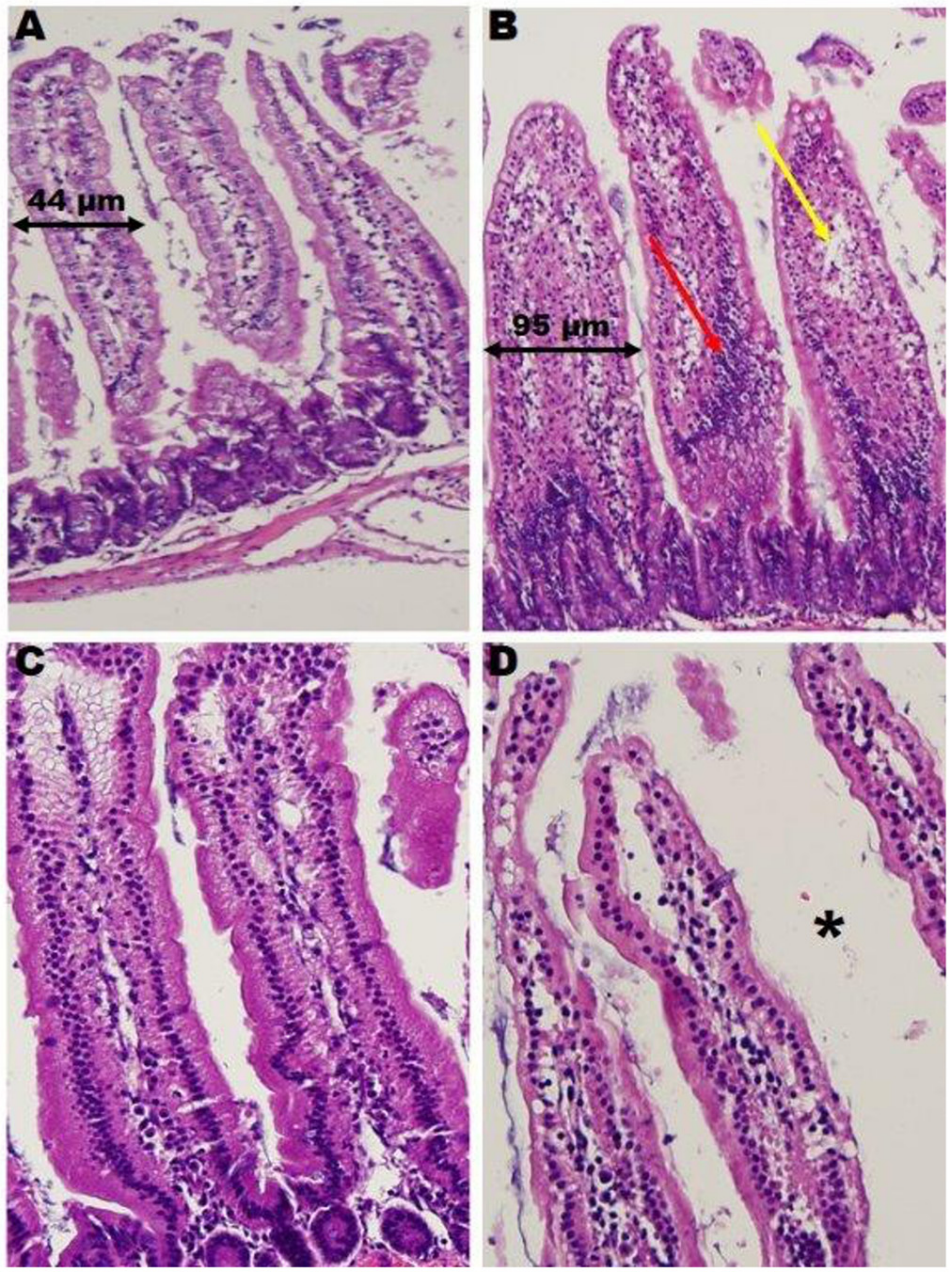
The Effects of Exercise on Duodenal Morphology. Widening in obese sedentary compared to lean sedentary animal (black arrows) due to: increase in inflammatory cells, plasmacystoid and lymphoid cells (red arrow); and infiltration of fat cells (yellow arrow). Exercise appears to protect the villi from widening. Obese exercise villi contain wider lumen (asterisk) and are devoid of fat cell infiltrate. Lean Sedentary (A), Obese Sedentary (B), Lean Exercise (C), Obese Exercise (D).

Sedentary Animals—The LS villous histology was normal, exhibiting a single layer of epithelium covering the villi and lamina propria. The OS villi were the same height as the LS (~300 μm tall) while the width was twice that of the LS animals (95 μm vs 44 μm, respectively) (Fig 1); as a result OS villi were crowded. Major causes of villi widening include an increase in inflammatory cells present in lymph and plasma cells and an increase in fat cells.

Exercised Animals—LX and OX villi were histologically normal with open lumens. The villi were well-formed and thicker than their sedentary counterparts, which was primarily due to vessel dilation. In the OX group, the plasmacystoid and lymphocytic infiltrate was absent in contrast to their sedentary counterparts. These results suggest that exercise prevented the morphological changes that were associated with high fat feedings and reduced inflammatory infiltrate.

#### Structural Distal Small Intestine Integrity (occludin and E-cadherin)

**Fig 2:**
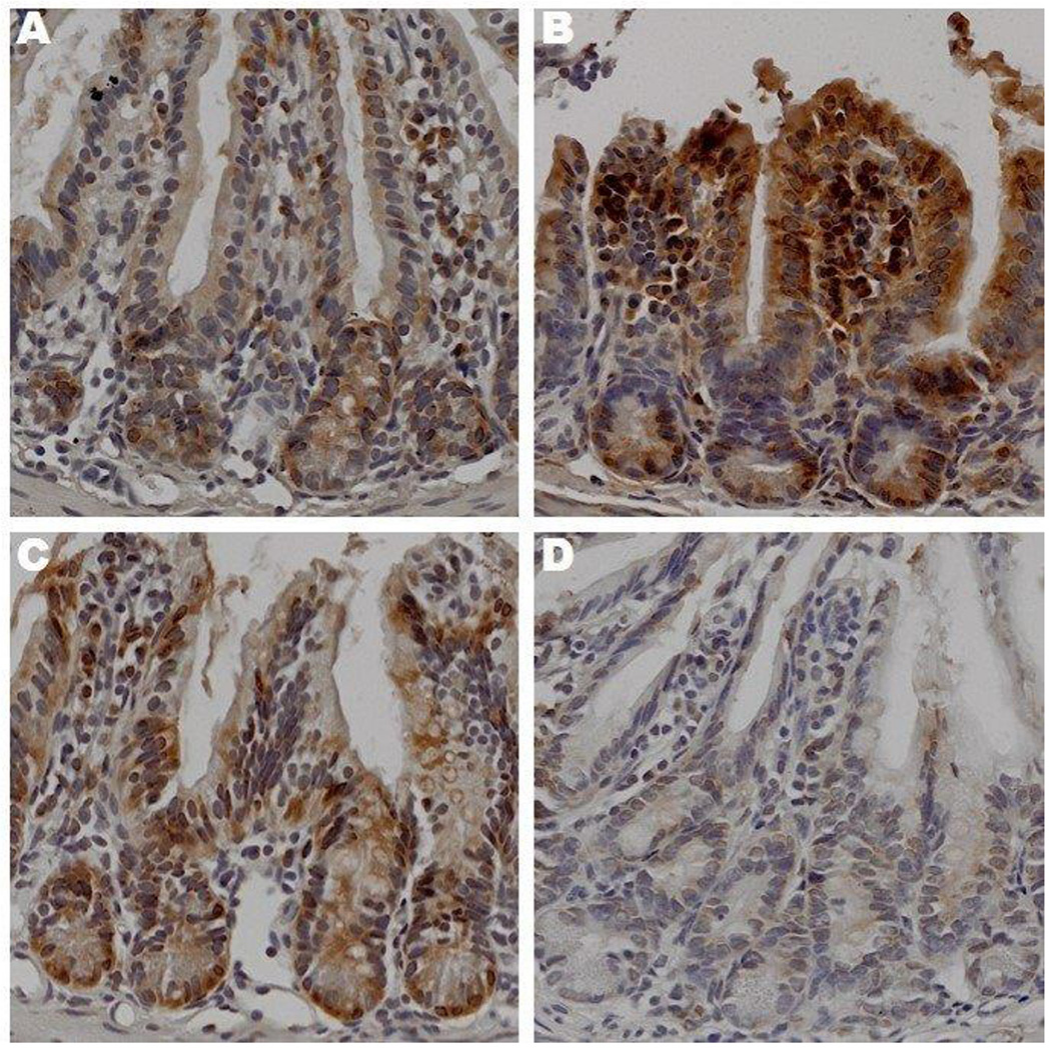
The Effects of Exercise on Occludin Expression in Ileum. Sections prepared after 12 weeks of treatment lean sedentary (A), obese sedentary (B), lean exercise (C), obese exercise (D),were stained with anti-occludin antibody. Binding was visualized using a Vectastain Elite ABC kit (original magnification x 400). One representative section from 6 mice/treatment group is shown.

**Fig 3:**
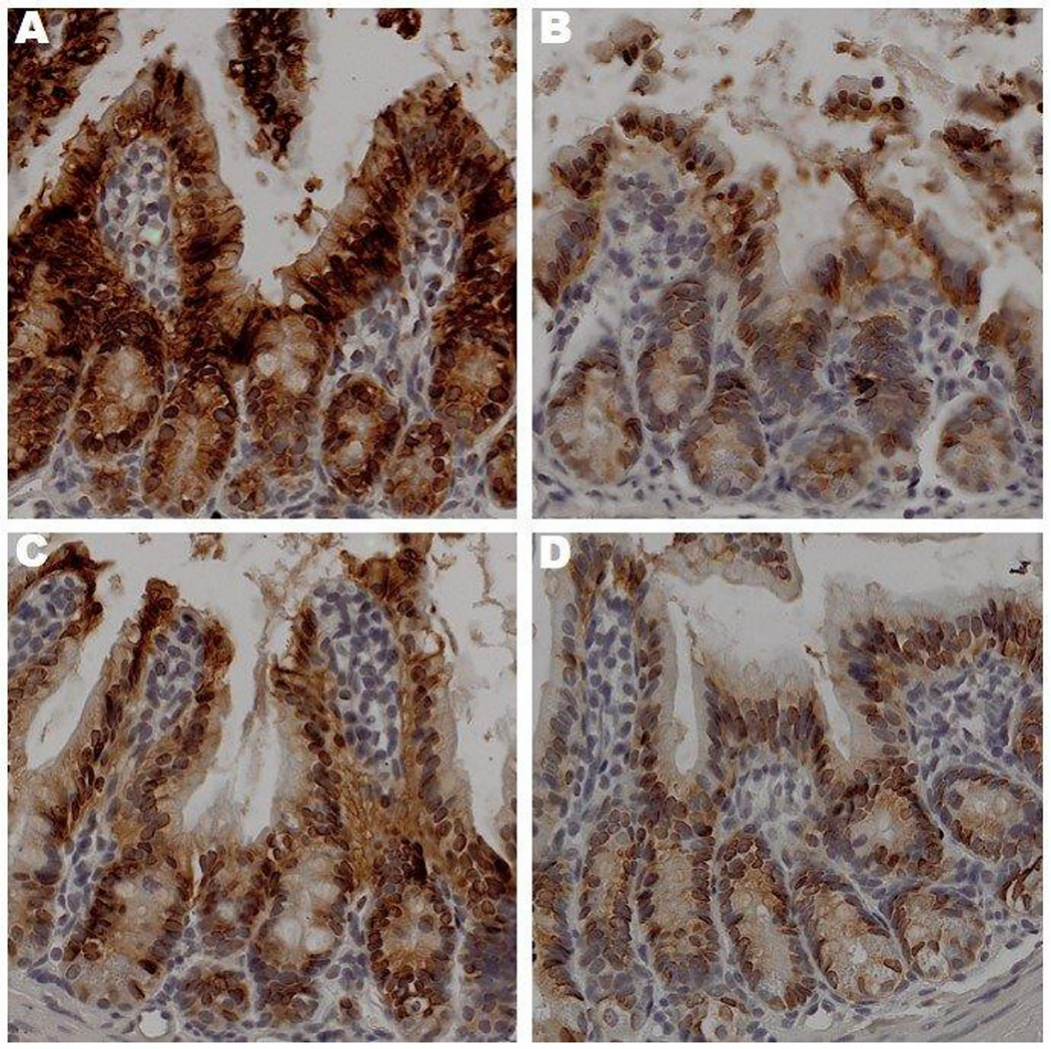
The Effects of Exercise on E-cadherin Expression in Ileum. Sections prepared after 12 weeks of treatment lean sedentary (A), obese sedentary (B), lean exercise (C), obese exercise (D),were stained with anti-E-cadherin antibody. Binding was visualized using a Vectastain Elite ABC kit (original magnification x 400). One representative section from 6 mice/treatment group is shown.

Sedentary animals—Occludin expression was up-regulated in the OS group compared to the LS (Fig 2). Contrary, E-cadherin expression was up-regulated in the LS group compared to the OS counterpart (Fig 3).

Exercised Animals—Occludin expression in the LX and LS mice was similar. Interestingly, occludin expression had decreased in the OX animals as compared to the LX, LS and OS groups (Fig 2). E-cadherin expression in the LX group was lower than its sedentary counterpart with lowest expression observed in the OX group (Fig 3). These results initially seem confusing as we expected occludin expression to be highest in the exercised groups, not the obese sedentary group. However, the higher E-cadherin expression in the groups with lower occludin expression supports the notion that barrier function is dynamic and that regulation of its function may not be dependent upon the protein expression but other factors like plasma membrane channels and transporters [23].

#### Exercise Reduces Intestinal Inflammation in Proximal and Distal Gut

**Fig 4:**
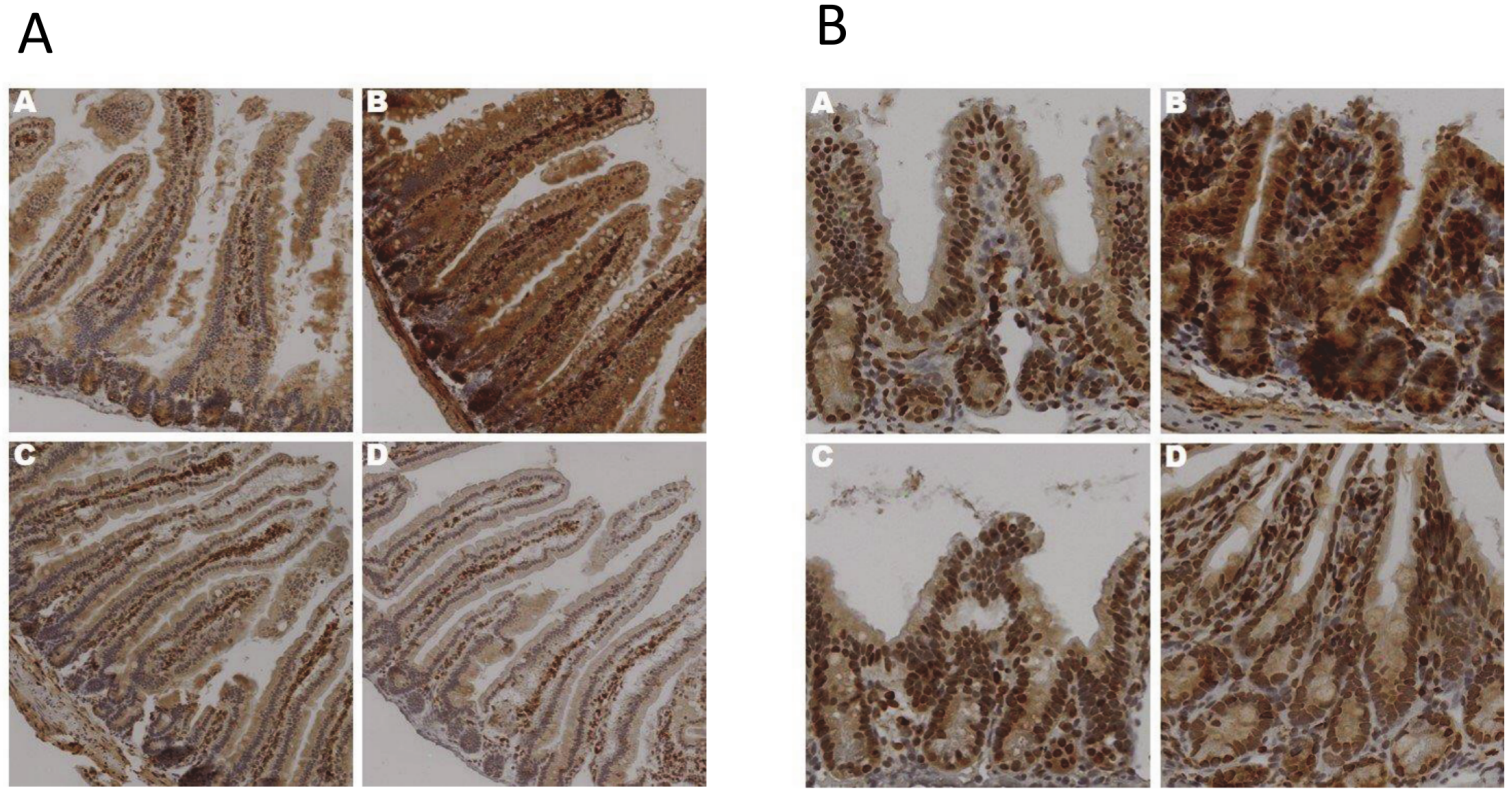
The Effects of Exercise on COX-2 Expression in Duodenum. Duodenal (a) and Ileal (b) sections prepared after 12 weeks of treatment lean sedentary (A), obese sedentary (B), lean exercise (C), obese exercise (D),were stained with anti-COX-2 antibody. Binding was visualized using a Vectastain Elite ABC kit (original magnification x 400). One representative section from 6 mice/treatment group is shown.

Sedentary animals—COX-2 expression was increased in the duodenum (Fig 4a) and ileum (Fig 4b) in the OS animals compared to the LS ones suggesting that high fat diets increased intestinal inflammation, which is in agreement with the H&E staining.

Exercised animals—Both LX and OX groups had COX-2 expression similar to the LS group. Furthermore, the OX group had less COX-2 expression compared to the sedentary counterpart, OS (Fig 4a and 4b). These data support the observed H&E findings that exercise reduced inflammation due to a high-fat diet.

#### Exercise Influences Gut Microbiota

**Fig 5:**
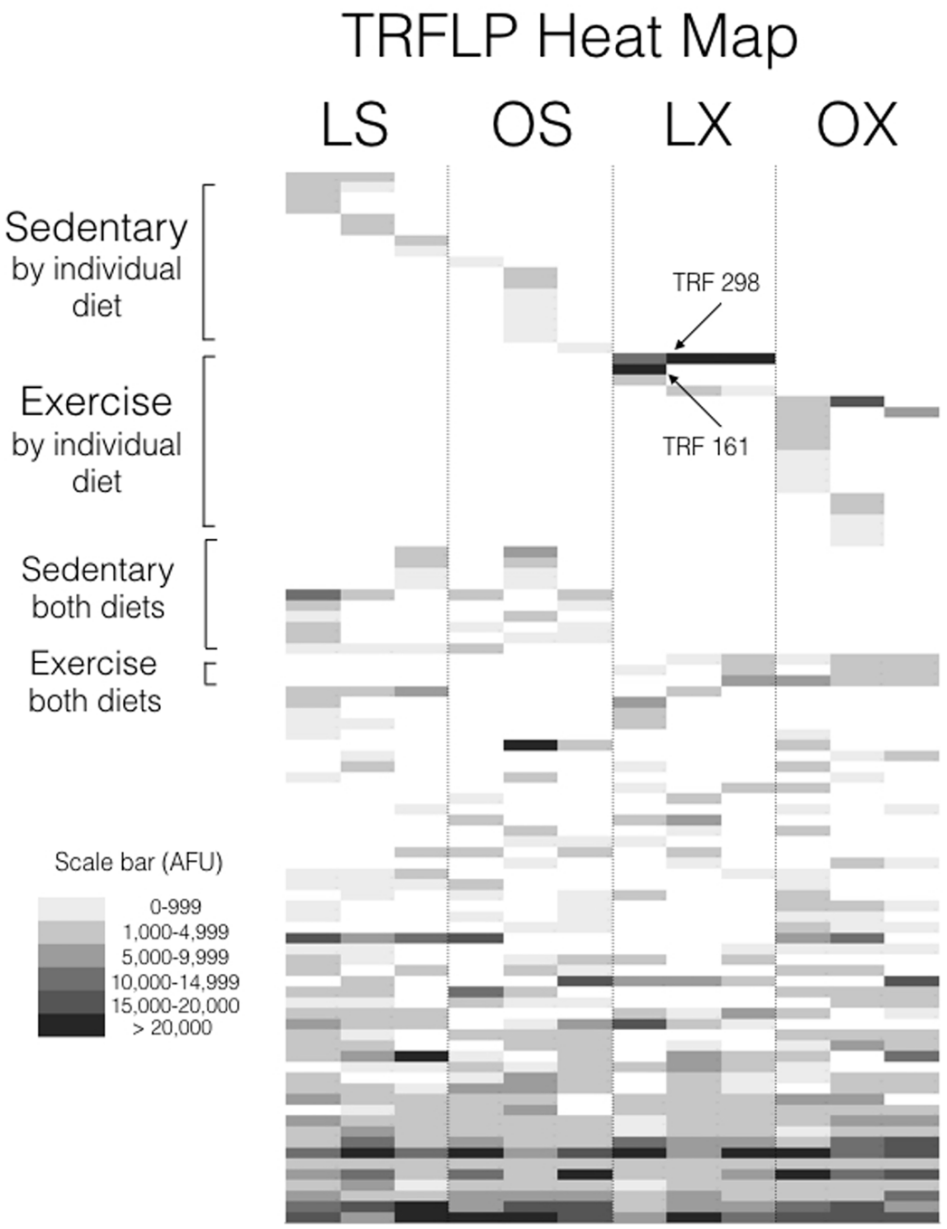
Heat Map. Heat map of average peak area within TRFLP profiles of fecal pellet bacterial community grouped by treatment (L, lean diet; O, high fat diet; S, sedentary; X, exercise). The major peaks in the lean exercise treatment are indicated.

A 16S rRNA gene analysis was performed and an example of the community profiles is shown in S1 Fig. Terminal restriction fragment length polymorphism (TRFLP) detected 100 peaks (OTUs) in all experimental treatments. A cluster analysis of the profiles of fecal pellets from individual animals demonstrated distinct groupings within the experimental treatments supported by bootstrap values >0.72 (S2 Fig). A heat map (Fig 5) of the average TRFLP peak areas indicated that 35 terminal restriction fragments (TRFs) were only found within a single experimental treatment, with approximately half of the unique TRFs observed in the normal diet/exercised animals (e.g. TRFs 298 and 161; Fig 5; S3 Fig) vs. the sedentary and exercised lean diet animals (S4 Fig). The other remaining TRFs were detected in nearly all the animals regardless of diet or exercise (S5 Fig). In order to identify specific bacterial 16S rRNA genes associated with the various TRFs, clone libraries were generated for all experimental groups (S1 Fig).

DNA from replicate samples were combined for pyrosequencing analysis which resulted in the detection of over 440 OTUs with 90% of the OTUs present at <0.1% within the whole sample set. A pie-chart of the top 20 OTUs (representing OTUs >1% of the sample set; S6 Fig) indicated that *Roseburia intestinalis*, *Allobaculum* spp., *Enterorhabdus* spp., and *Blautia* spp. were the predominant bacteria observed in all groups. *Allobaculum* spp. and *Clostridiales* were enriched within the exercised groups.

**Fig 6:**
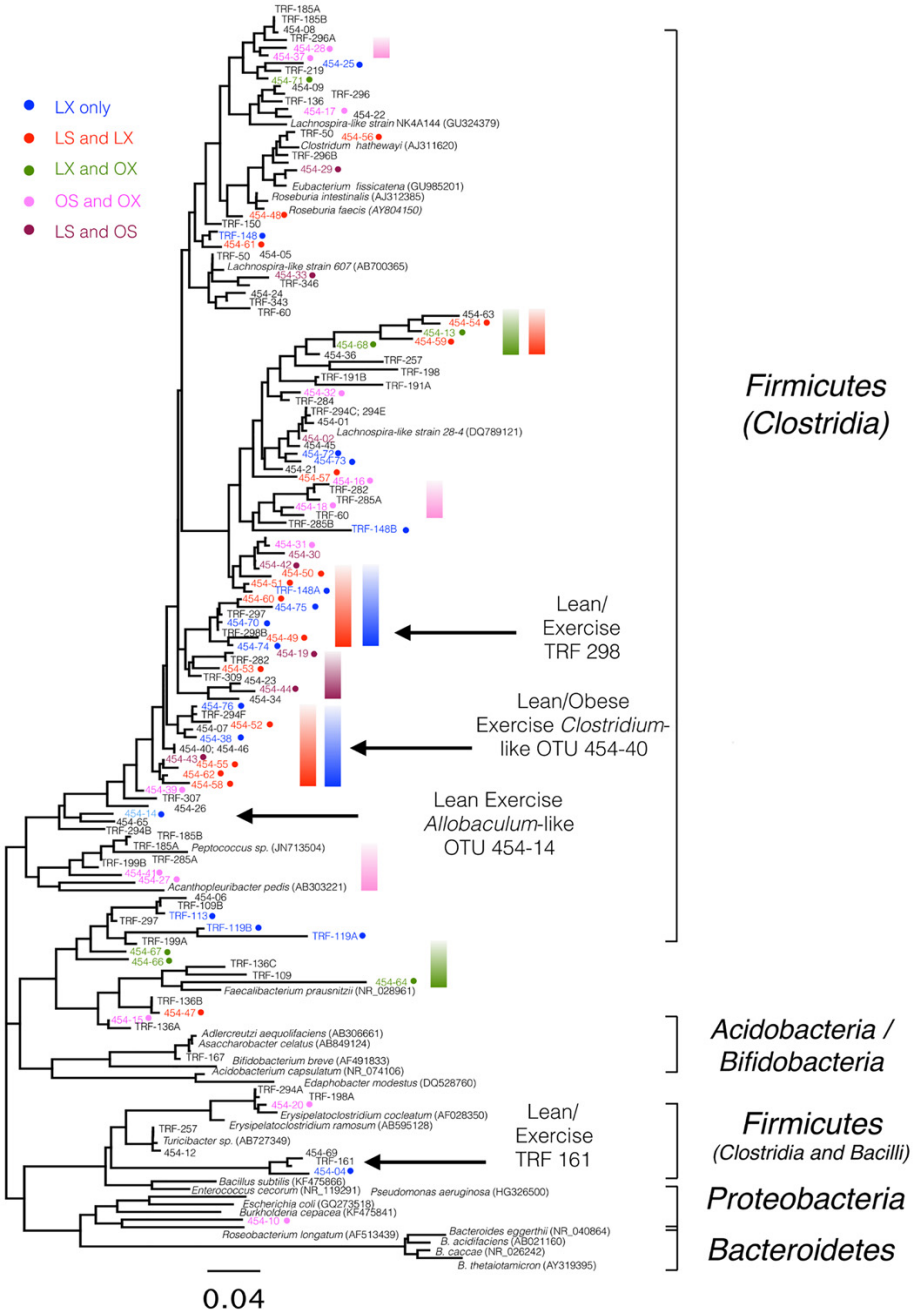
Phylogenetic Tree. Maximum likelihood phylogenetic tree from TRFLP/clones and pyrosequencing based on 379 unambiguously aligned bases. Color coding for the experimental treatments is indicated.

A phylogenetic analysis of the 76 OTUs detected by pyrosequence analysis and the 57 TRFLP clones demonstrated broad agreement (Fig 6). Both the clonal and pyrosequencing analyses revealed that few *Bacterioides* family members were observed in the fecal microbiota. Rather, *Clostridiales* dominated the fecal microbiota and this was observed in all animal groups. Interestingly, *Faecalibacterium prausnitzi* was only detected in exercised animals (green bar), while animals on the normal diet, regardless of exercise, had large clusters of sequences related to *Lachnospiraceae* spp. that were not present in the high fat fed animals (blue and red bars). *Allobaculum* spp. and *Clostridium* spp. were enriched in the exercise animals on a normal diet (LX) whereas the high fat fed animals (OS and OX) had microbial clusters related to *Peptococcus* spp. (pink bar).

### Blood Profiles of Sedentary and Exercised Animals on Normal and High-Fat Diets

Lean sedentary animals had lower levels of IL-6 and insulin compared to the obese sedentary counterparts (Table 2). Furthermore, exercised animals showed lower ghrelin levels compared to both sedentary groups. In addition, the exercise groups had lower IL-6 compared to the OS group but higher values than the LS group and were not significantly different from each other. Both PYY and PP was highest in the LX group followed by the OX group and these values were higher compared to both sedentary groups. Insulin values were lowest in the LX group and highest in the OS group with LS and OX being similar and not different from each other, but significantly lower that OS. Finally, amylin values were higher in both exercise groups compared to sedentary. OX had a significantly higher amylin concentration compared to LX.

**Table 2:** Plasma Systemic Biomarkers.

Biomarker (pg/mL)*	Lean-Sedentary	Lean-Exercise	DIO-Sedentary	DIO-Exercise
Ghrelin	84.0±3.4^a^	33.8±4.5^b^	87.4±4.1^a^	32.1±6.3^b^
IL-6	22.0±0.7^a^	39.0±7.2^b^	85.5±3.8^c^	31.0±5.6^b^
Insulin	13.1±5.2^a,c^	7.1±2.5^a^	31.0±4.1^b^	15.7±6.0^c^
PYY	8.9±3.7	36.0±9.6	10.8±6.4	25.7±3.6
PP	5.4±1.7^a^	19.6±7.3^b^	7.6±6.9^a^	15.2±4.3^b^
Amylin	25.0±0.7	43.4±7.2^a^	26.8±2.7	71.1±5.1^b^

*Values are mean ± SEM, n = 9 in each group.

Values that do not share the same superscript letters are significantly (*p<0*.*05*) different from each other.

## Discussion

**Fig 7:**
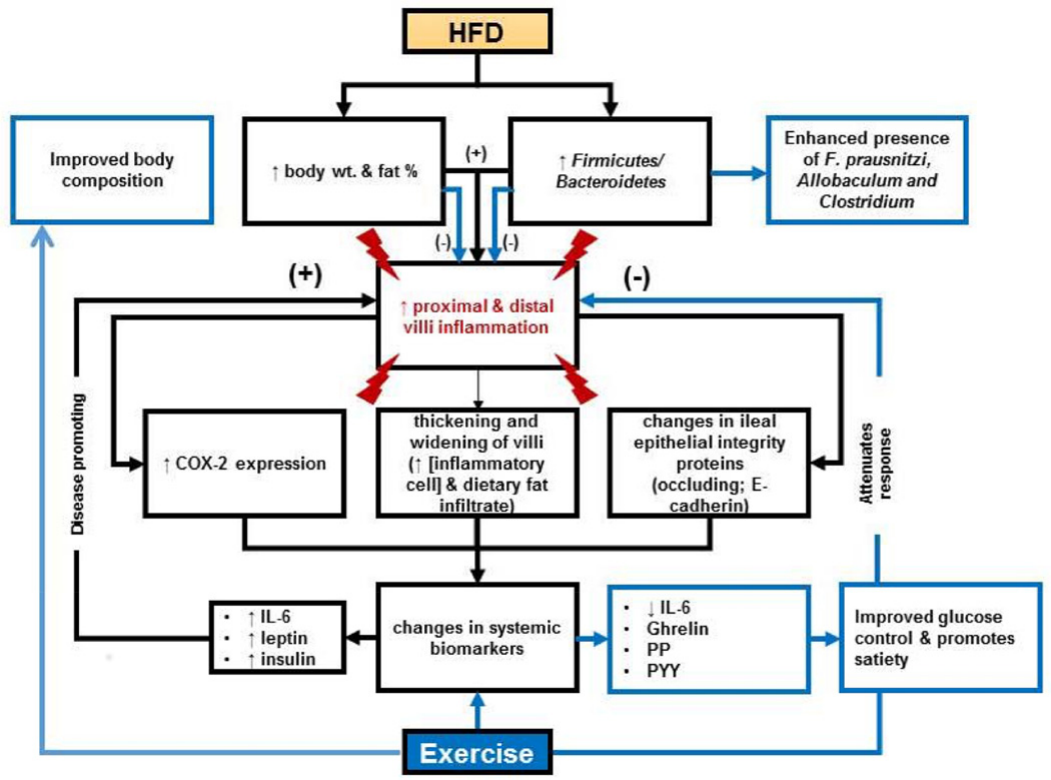
Experimental Results Summary. Impacts of high-fat diet (HFD) and exercise on intestinal tissue, microbiome and systemic biomarkers. Results indicate exercise can protect intestinal morphology in the presence of a HFD, promote a diverse microbiome that has microbes that promote intestinal health and reduce systemic inflammation while promoting satiety.

The major findings of these studies indicate that: (1) high-fat diets altered intestinal morphology particularly of the duodenum; (2) exercise protected duodenal morphology in the presence of a high-fat diet; (3) high-fat diets increased intestinal inflammation and exercise reduced it; (4) exercise manifested a unique microbiome independent of diet; (5) exercise reduced blood levels of IL-6, insulin and ghrelin and increased levels of satiety related hormones (Fig 7). We observed that high fat diets accompanied with sedentary behavior increased the width of duodenal villi. We are the first using IHC to substantiate *in situ* inflammation and loss of intestinal integrity due to high fat diet and sedentary lifestyle in mice. These data correlate with results from others in that animals fed a high-fat diet had a three-fold increase in TG accumulation in intestinal mucosa and an up-regulation of genes for TG synthesis, chylomicron secretion and uptake, oxidation and de novo synthesis of fatty acids [47]. These observations, accompanied with an impaired oral fat tolerance, suggest a reduced rate of intestinal lipid secretion led to increases in mucosal TG accumulation and contributed to the shortening and widening of the villi. Analysis of H&E stained tissue revealed significant amounts of lipid infiltration, inflammatory lymphocytes, plasma cells and macrophages. This was further supported by the increased expression of COX-2 in the high fat fed sedentary animals. An important finding of our work suggests that exercise prevented high-fat triggered morphological changes by reducing COX-2 expression in both proximal and distal gut.

The high fat diet induced changes in intestinal morphology and inflammation were negated by exercise, however, mechanisms associated with these observations need to be elucidated. There are a couple of plausible accepted explanations related to lipid metabolism for these observations; 1) regular exercise is associated with reduced postprandial lipemia; and 2) systemic metabolic adaptations to promote greater reliance on fat utilization during exercise and at rest [48,49]. Cross-sectional and longitudinal studies show that regular aerobic exercise reduced postprandial lipemia in the presence or absence of weight loss which may be intensity and dose-dependent (extensively reviewed in [50]). Exercise reduced postprandial lipemia by 3 possible mechanisms acting alone or in combination: (1) decreased appearance of chylomicron-TG concentrations from the gut, (2) increased clearance of TG-rich lipoproteins (VLDL and/or CM) via exercise- mediated increases in skeletal muscle and/or adipose tissue LPL activity, and (3) decreased hepatic VLDL-TG secretion (for review see [51]). Regardless of the substrate used during the exercise bout, the hepatic and skeletal muscle program following exercise is suggested to preferentially use fatty acids from TRLs to replenish ATP, thus sparing glucose [48,49]. Furthermore, in our studies there was a reduction of body weight in the high fat fed exercised animals. thus the energy deficit created by exercise appears to be a primary mediator of the exercise-induced decline in postprandial lipemia. These explanations may provide a rationale for normal morphology of the villi in the high fat fed exercised animals, suggesting that exercise promoted reduced postprandial lipemia and alterations in substrate use that favored fatty acids utilization to replenish energy stores between the exercise bouts.

Another plausible explanation for why exercise reduced intestinal inflammation is that exercise has been shown to increase antioxidant enzymes (glutathione peroxidase and catalase), anti-inflammatory cytokines (IL-10), and anti-apoptotic proteins (Bcl-2) in intestinal lymphocytes [39,40]. It was also observed that exercise decreased TNF-α, pro-apoptotic proteins (caspase 3 and 7) and the pro-inflammatory cytokine IL-17, suggesting that exercise can modulate the intestinal immune response [39,40]. These data correlate with our studies suggesting that exercise, through laminar shear stress activation, may decrease superoxide anion production, which in turn decreases ROS (reactive oxygen species) generation, and preserves endothelial NO bioavailability [52–55].

Occludins are integral membrane proteins crucial for tight junctions and the adherens are involved in cell to cell adhesion as well as communication [23]. Our studies showed that high-fat fed animals had reduced E-cadheren expression while interestingly the opposite was true for occludin. The occludin response differs from previous published data, suggesting impaired barrier function with high-fat diets [10,12]. One animal in the OX group had influenced our thoughts on these results. This animal’s exercise volume was only 50% of the other animals in the group and weighed 48 g at sacrifice. We observed that this mouse’s COX-2 and occludin expression was up-regulated compared to the other mice and interestingly E-cadherin expression was also up-regulated compared to the animals which exercised “normally”. These data suggest that the increased expression of occludin may not imply intact tight junctions but may be compensatory expression due to inflammation (COX-2) induced damage (S7 Fig). Research on exercise and barrier function suggests that the more strenuous the exercise the greater barrier disruption, due to changes in blood flow leading to insufficient removal of metabolites and delivery of nutrients [36]. Though the exercise protocol in these studies is not considered strenuous; investigation of blood flow changes, particularly in the obese gut, would be informative as there is a scarcity of literature in this area. Further, claudins play a critical role in barrier function by sealing neighbor epithelial cells and these were not examined. Information about their expression may help to fully understand the intricate relationship of the epithelial barrier.

Finally, the microbiome findings in this study appear to be similar (with some distinct differences) to a separate study conducted by Evans et al. on exercise and diet in mice [4]. Primarily, it was observed in both studies that exercise in the presence of a high fat diet (60% in the Evans study and 40% in the current study) maintains body weight with feedings [4]. Likewise, both studies demonstrated clustering of the microbial communities by experimental treatment using a TRFLP approach, despite the differences in DNA extraction procedures, PCR priming sets, and the restriction enzymes being used. However, Evans et al. [4] demonstrated that both the lean exercise and obese sedentary groups had statistically higher abundance of Bacteroidetes and lower abundance of Firmicutes. In contrast, Firmicutes were detected within the fecal samples from the high fat fed mice, which may be explained by the 2 different sources of mice (Jackson Laboratories and Taconic Laboratories), the differences in DNA extraction (bead beating/12 hrs at 55°C vs. 5 freeze/thaws and immediate extraction), or the use of different primers (group specific vs. universal primers) for amplifying the target 16S rRNA genes. Additionally, in our study the microbiota was analyzed at the genus level rather than the phylum/class or family level. Our findings which are in agreement with Khan et al. [56], suggesting that bacteria related to *Faecalibacterium prausnitzi* are present in exercised mice and may provide protection to the gut through oxygen detoxification by a flavin/thiol electron shuttle in *F*. *prausnitzi*. Furthermore, the integrity of gut immunological markers observed in our study is consistent with the hypothesis that *F*. *prausnitzi* promotes a healthy digestive tract by producing butyrate and lowering the oxygen tension in the lumen as described in humans [57]. *Lachnospiraceae*, as a cluster of *Clostridia*, in the exercise groups alone (LX and OX; Fig 7; blue/red bars) contrast the sedentary group which had different set of clostridia related to *Lachnospiraceae* (Fig 7; pink bars). The concept that various *Lachnospiraceae* may be beneficial to the gut while other closely related strains are not beneficial is supported by a study of 30 *Lachnospiraceae* genomes, where less than half of the strains were found to possess the genes for butyrate production [58]. Our data suggests that further examination of the physiology of distinct *Clostridiales* species within the mouse gut will be needed to resolve the role of butyrate production or other mechanisms in the promotion of a healthy gut microbiome.

The mouse blood work results suggest that high fat diets promote systemic inflammation as evident by the elevated plasma IL-6. While IL-6 is often used as a marker for obesity-associated ‘meta-inflammation [26]’ measurement of plasma endotoxin levels (LPS), which were not assessed would be ideal and should be done in future studies. Of note, our lean exercise animals had higher levels of IL-6 compared to the sedentary counter parts and this may be related to the duality of IL-6 function. It is accepted that exercising skeletal muscle produces IL-6 and that this net release from muscle can increase circulating concentrations leading to hepatic glucose output and lipolysis [58]. This may indicate that there may be a link to IL-6 released by exercising muscle and exercise-related metabolic changes. Furthermore, high fat fed animals had higher levels of insulin and leptin, which are common for this model and exercise regulated this along with hormones of satiety and glucose control.

These studies support the hypothesis that exercise manifests a unique microbiome independent of diet. Exercise reduced the intestinal inflammatory response due to high-fat diet which lead to morphology similar to the sedentary animals and promoted satiety biomarkers. Taken together these data suggest that exercise is a safe and efficacious strategy to combat obesity through positive changes in microbial ecology and intestinal health.

## Supporting Information

S1 Fig Example TRFLP profiles of fecal pellet bacterial community. The biological replicate for each treatment is indicated. Those TRFLP peaks represented in clone libraries from each treatment are highlighted in black.
(TIFF)

S2 Fig Cluster analysis of replicate TRFLP profiles from the different experimental treatments (L, lean diet; O, high fat diet; S, sedentary; X, exercise). The numbers indicate bootstrap support for the groupings.
(TIFF)

S3 Fig Histogram of TRF average peak areas found in 1 diet/treatment as indicated. Error bars indicate standard deviation of the biological replicates in positive direction only. Note the difference in vertical scale.
(TIFF)

S4 Fig Histogram of TRF average peak areas found in 2 diet/treatments as indicated. Error bars indicate standard deviation of the biological replicates in positive direction only.
(TIFF)

S5 Fig Histogram of TRF average peak areas for all diet/treatments. Error bars indicate standard deviation of the biological replicates in positive direction only.
(TIFF)

S6 Fig Pyrosequencing analysis of the different microbial communities from the experimental treatments. The OTUs in the exercise treatment that are nearly undetectable in the sedentary treatment have been pulled away from the center. Only those OTUs greater than 4% have values shown and can be read clockwise to coordinate with the key.
(TIFF)

S7 Fig The Effects of Minimal Exercise on Ileum in Obese Gut. Section was prepared after 12 weeks of feeding a high-fat diet and limited usage of free running wheel (exercise volume was 50% less than animals in this cohort). Binding was visualized using a Vectastain Elite ABC kit (original magnification x 400). Representative section is shown COX-2 (A), Occludin (B), E-Cadherin (C).
(TIFF)

S1 Table Pyrosequences used in the phylogenetic analysis. (DOCX)
