# Supplementary figures and images for: The Effect of Diet and Exercise on Intestinal Integrity and Microbial Diversity in Mice
Source: PLoS One. 2016 Mar 8;11(3):e0150502. doi: 10.1371/journal.pone.0150502 (PMC4783017; doi:10.1371/journal.pone.0150502)

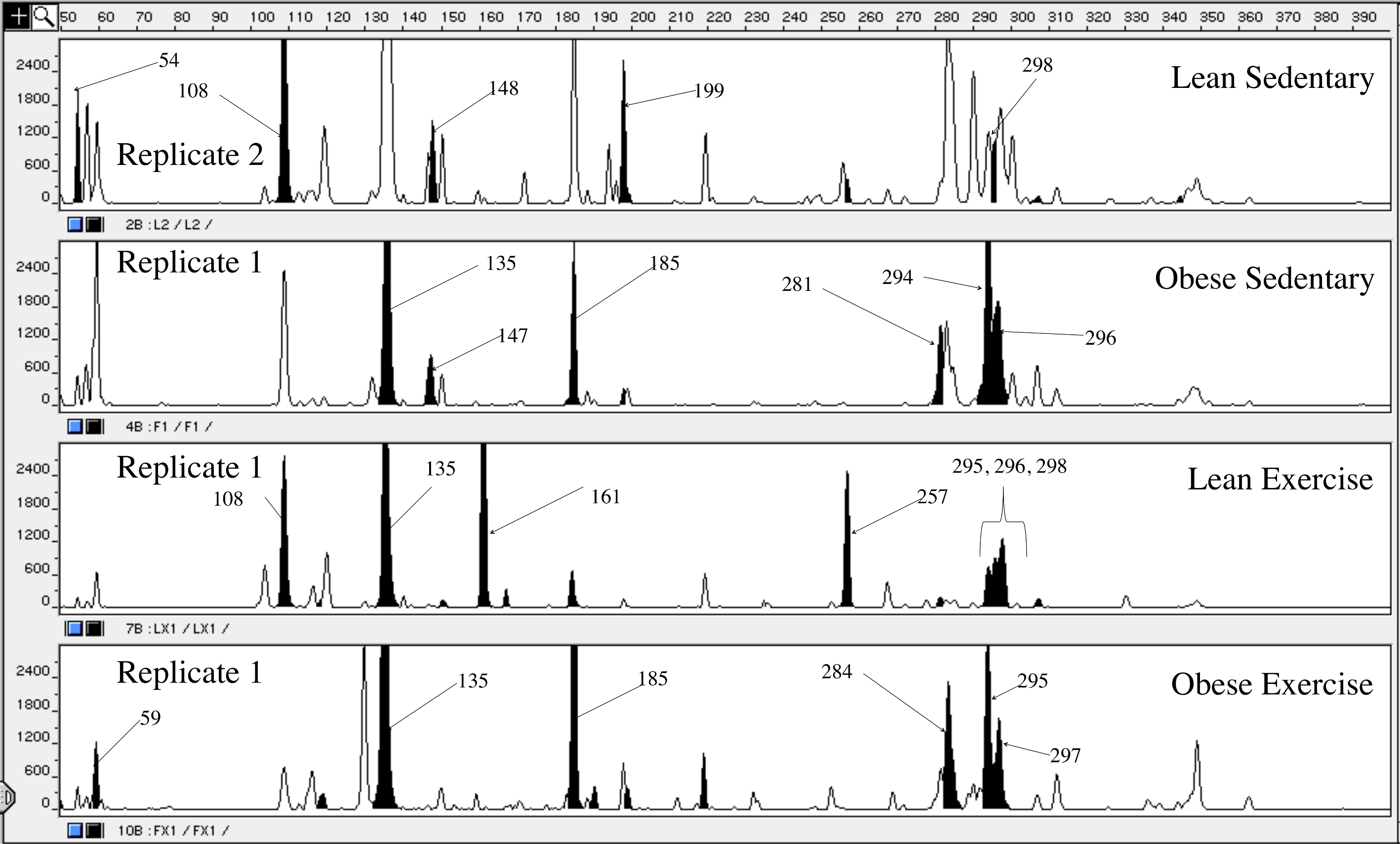

Supplement: S1 Fig — The biological replicate for each treatment is indicated. Those TRFLP peaks represented in clone libraries from each treatment are highlighted in black. (TIFF) [file pone.0150502.s001.tiff]

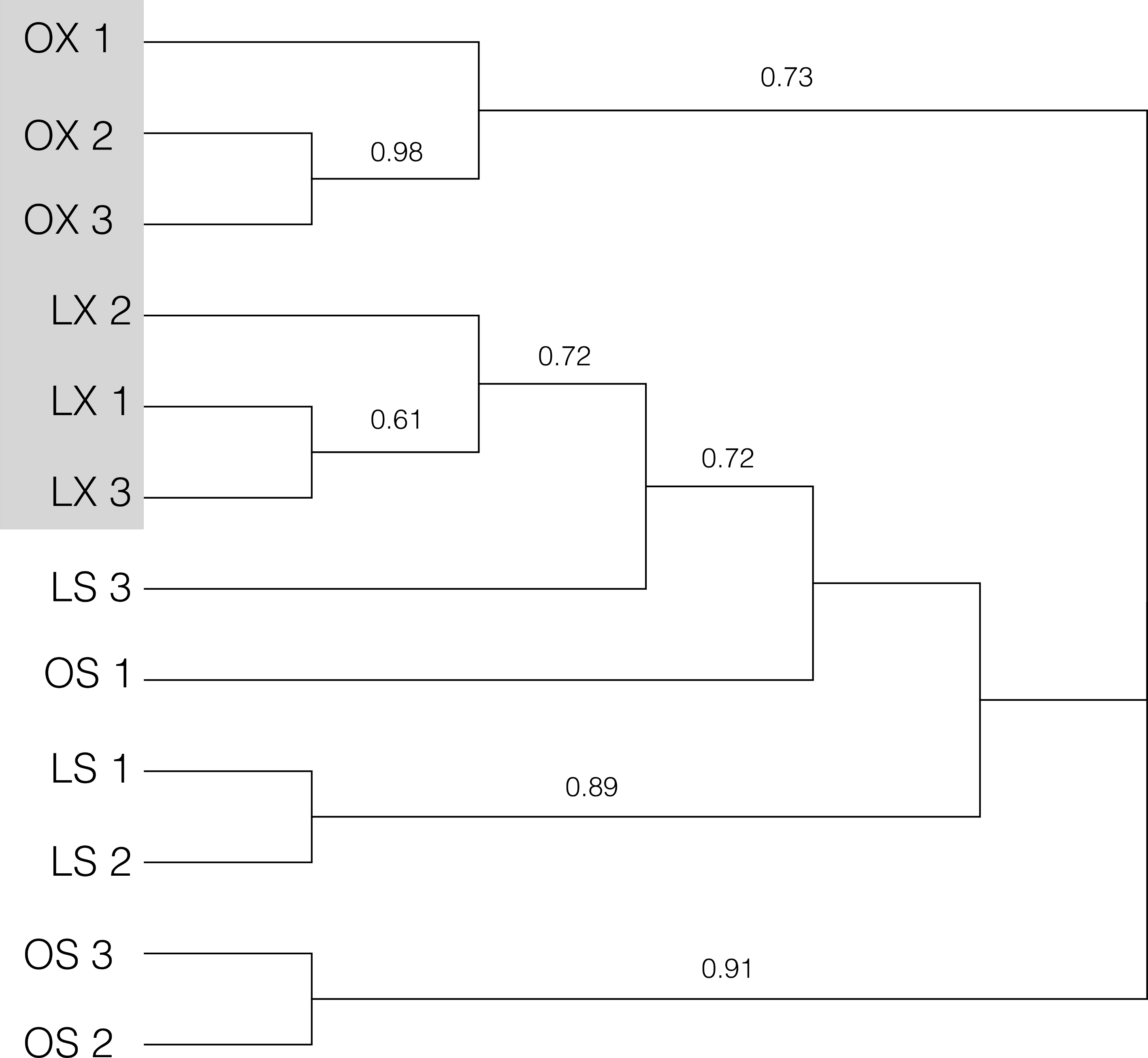

Supplement: S2 Fig — The numbers indicate bootstrap support for the groupings. (TIFF) [file pone.0150502.s002.tiff]

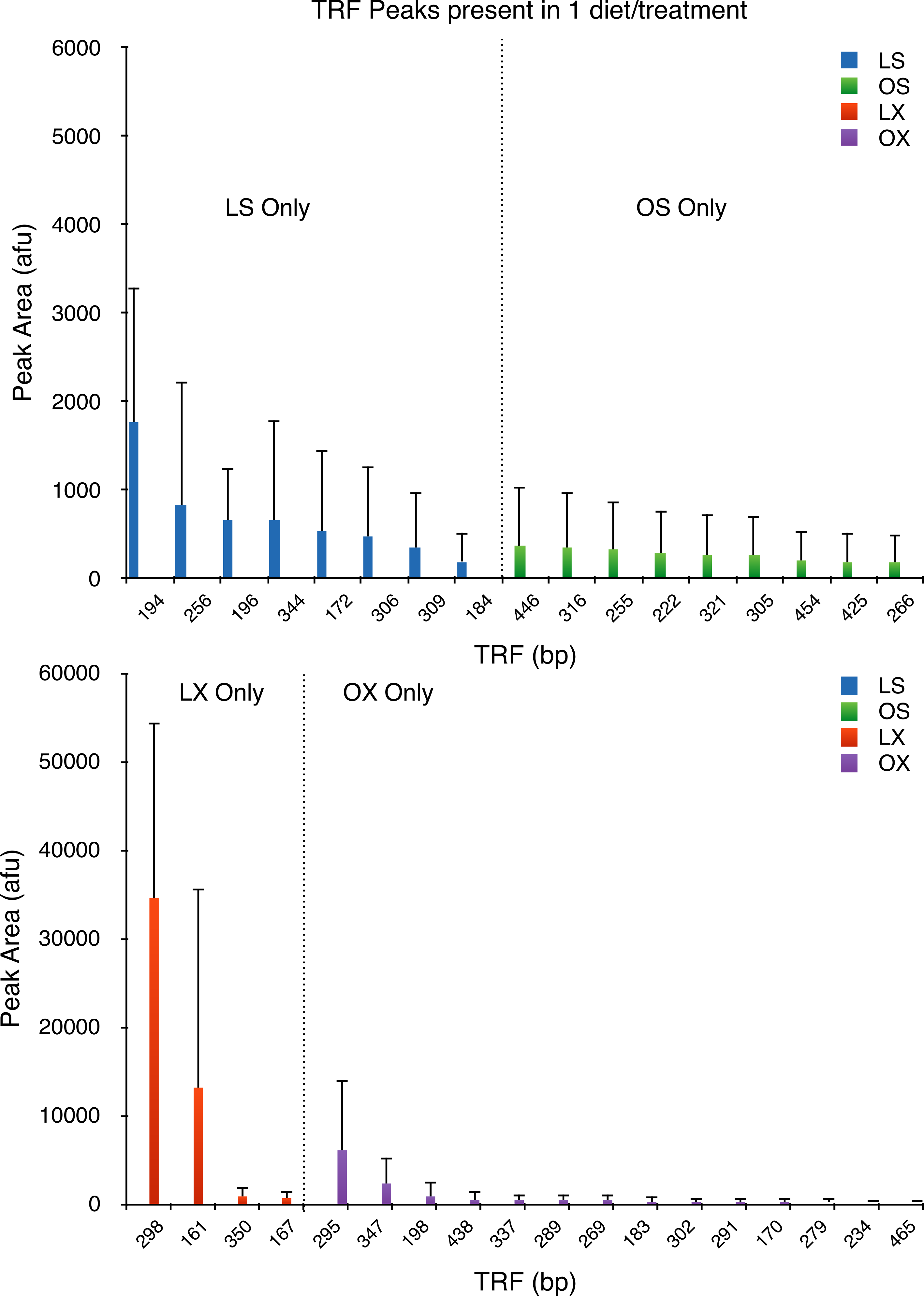

Supplement: S3 Fig — Error bars indicate standard deviation of the biological replicates in positive direction only. Note the difference in vertical scale. (TIFF) [file pone.0150502.s003.tiff]

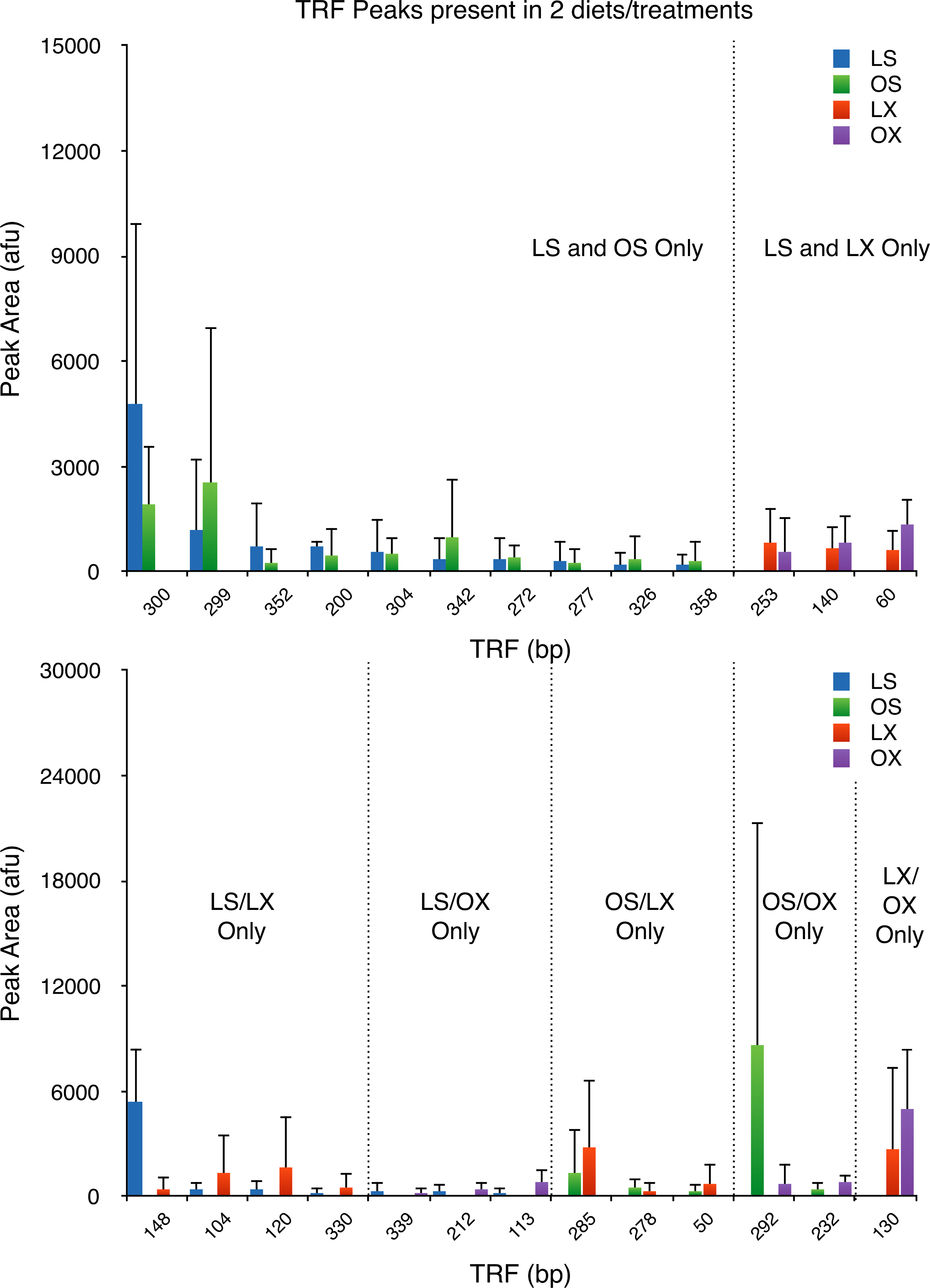

Supplement: S4 Fig — Error bars indicate standard deviation of the biological replicates in positive direction only. (TIFF) [file pone.0150502.s004.tiff]

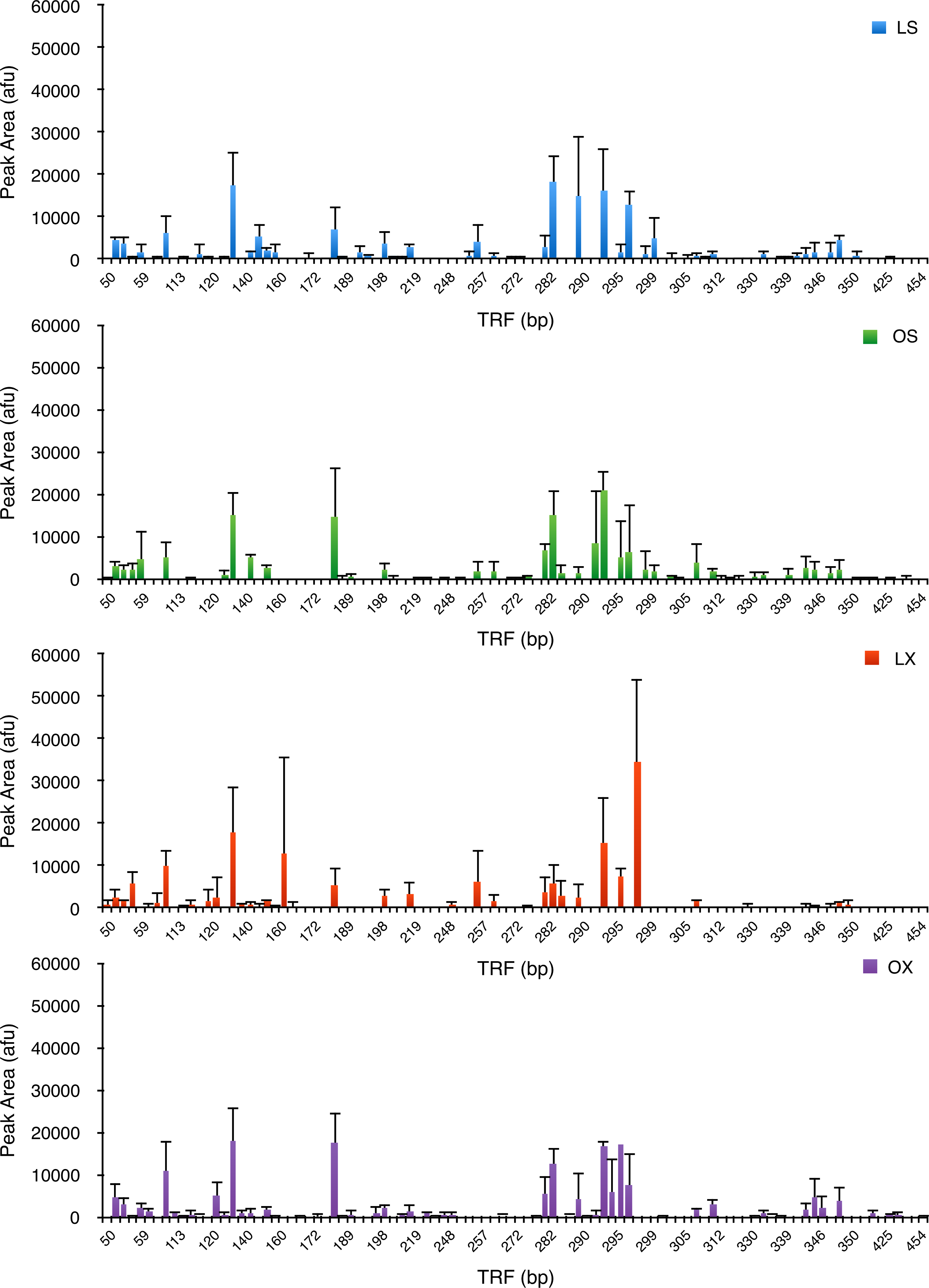

Supplement: S5 Fig — Error bars indicate standard deviation of the biological replicates in positive direction only. (TIFF) [file pone.0150502.s005.tiff]

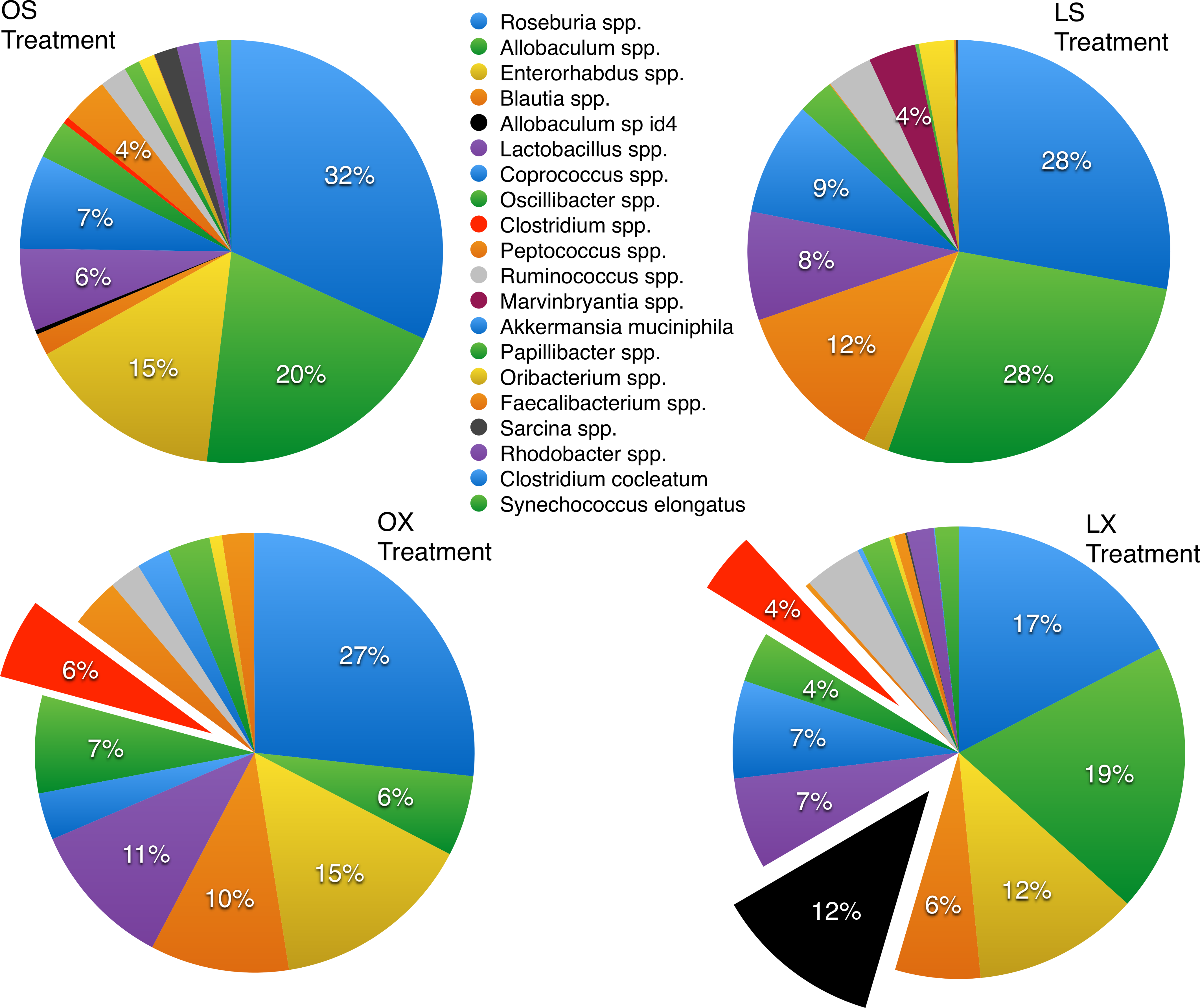

Supplement: S6 Fig — The OTUs in the exercise treatment that are nearly undetectable in the sedentary treatment have been pulled away from the center. Only those OTUs greater than 4% have values shown and can be read clockwise to coordinate with the key. (TIFF) [file pone.0150502.s006.tiff]

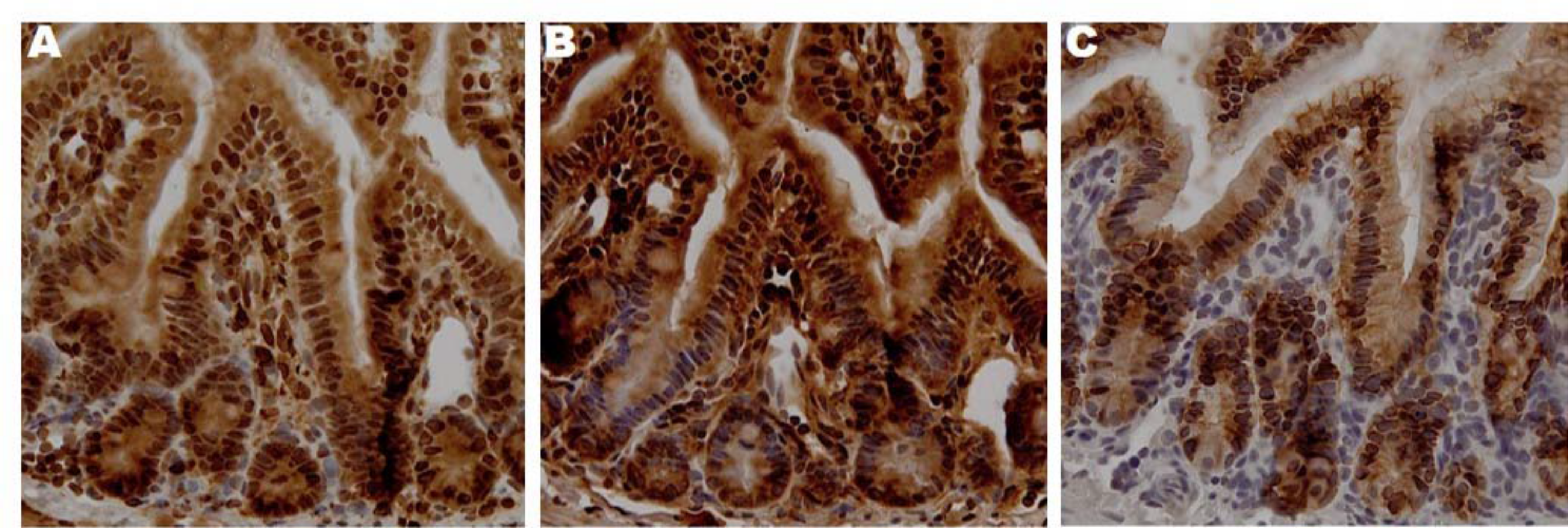

Supplement: S7 Fig — Section was prepared after 12 weeks of feeding a high-fat diet and limited usage of free running wheel (exercise volume was 50% less than animals in this cohort). Binding was visualized using a Vectastain Elite ABC kit (original magnification x 400). Representative section is shown COX-2 (A), Occludin (B), E-Cadherin (C). (TIFF) [file pone.0150502.s007.tiff]
