# Supplementary material for: The Effect of Diet and Exercise on Intestinal Integrity and Microbial Diversity in Mice
Source: PLoS One. 2016 Mar 8;11(3):e0150502. doi: 10.1371/journal.pone.0150502 (PMC4783017; doi:10.1371/journal.pone.0150502)
Supplement: S1 Table — (DOCX) [file pone.0150502.s008.docx]

>LK5::ISJPSFI02F00JM AOTU007 454-01

TATAAGAGAGGGTTTGATCCTGGCTCAGGATGAACGCTGGCGGCGTGCCTAACACATGCAAGTCGAACGGGTGTACGGGGAGGAAGGCTTCGGCCGGAAAACCTGTGCATGAGTGGCGGACGGGTGAGTAACGCGTGGGCAACCTGGCCTGTACAGGGGGATAACACTTAGAAATAGGTGCTAATACCGCATAACGGGGGAAGCCGCATGGCTTTTCCCTGAAAACTCCGGTGGTACAGGATGGGCCCGCGTCTGATTAGCCAGTTGGCAGGGTAACGGCCTACCAAAGCGACGATCAGTAGCCGGCCTGAGAGGGCGGACGGCCACACTGGGACTGAGACACGGCCCAGACTCCTACGGGAGGCAGCAGTGGGGGATATTGCACAATGGGGGGAACCCTGATGCAGCGACGCCGCGTGGGTGAAGAAGCGCCTCGGCGCGTAAAGCCCTGTCAGCAGGGAGAAAATGACGGTACCTGAAGAAAAAGCCCCGGCTAACTACGTGCCAGCAGCCGCGGTAAT

>LK5::ISJPSFI02F23Q0 5OTU009 454-02

TATAAGAGAGCGTTTGATCATGGCTCAGGATGAACGCTGGCGGCGTGCCTAACACATGCAGTCGAACGGGTGTACGGGATGGAAGATTTCGGTCGGAAGCCCCGTGCATGAGTGGCGGACGGGTGAGTAACGCGTGGGCAACCTGCCCTGTACAGGGGGACAACACTTAGAAATAGGTGCTAATACCGCATAACGGGGGGAGCCGCATGGCTTCCCCCTGAAAACTCCGGTGGTACAGGATGGGCCCGCGTCTGATTAGCCAGTTGGCAGGGTAACGGCCTACCAAAGCGACGATCAGTAGCCGGCCTGAGAGGGCGGACGGCCACACTGGGACTGAGACACGGCCCAGACTCCTACGGGAGGCAGCAGTGGGGGATATTGCACAATGGGGGGAACCCTGATGCAGCGACGCCGCGTGGGTGAAGAAGCGCCTCGGCGCGTAAAGCCCTGTCAGCAGGGAAGAAAATGACGGTACCTGAAGAAGAAGCCCCGGCTAACTACGTGCCAGCAGCCGCGGTAA

>LK5::ISJPSFI02FRAU0 5OTU011 454-03

TATAAGAGAGGGTTTGATCCTGGCTCAGGACGAACGCTGGCGGCGTGCCTAACACATGCAAGTCGAACGAAGCTTGACGAATGATTTCTTCGGAATGAAATCTGATATGACTGAGTGGCGGACGGGTGAGTAACGCGTGAGCAACCTGCCCTTCGGAGGGGGATAGTGTCTGGAAACGGACAGTAATACCGCATAACGTATTTTTACCGCATGATAGAAATACCAAAACTGAGGTGCCGAAGGATGGGCTCGCGTTGGATTAGATAGTTGGTGGGGTAACGGCCTACCAAGTCGACGATCCATAGCCGGACTGAGAGGTTGAACGGCCACATTGGGACTGAGACACGGCCCAGACTCCTACGGGAGGCAGCAGTGGGGAATATTGCACAATGGGGGAAACCCTGATGCAGCAACGCCGCGTGAAGGAAGACGGTTTTCGGATTGTAAACTTCTGTTCTTAGTGAAGAATAATGACGGTAGCTAAGGAGCAAGCCACGGCTAACTACGTGCCAGCAGCCGCGGTAATT

>LK5::ISJPSFI02G4UAY 5OTU014 454-04

TATAAGAGAGAGTTTGATCCTGGCTCAGGATGAACGCTGGCGGCATGCCTAATACATGCAAGTCGAACGGGAGACCTTCGGGTCTCTAGTGGCGAACGGGTGAGTAACACGTAGGGAACCCGCCCGCGCACCGGGAATACGCTCTGGAAACGGAGAACAAATCCCGATGTACAGGAAGGAGGCATCTTCTTTCTGTGAAACATCCTTTAGGGGATGGGGCGCGGATGGACCTGCGGTGCATTAGTTGGTTGGCGGGGTAAAGGCCCACCAAGACGATGATGCATAGCCGGCCTGAGAGGGCGGACGGCCACATTGGGGCTGAGACACGGCCCAGACTCCTTCGGGAGGCAGCAGTAGGGAATTTTCGTCAATGGGCGCAAGCCTGAACGAGCGATGCCGCGTGAGTGAAGAAGGCCTTCGGGTCGTAAAGCTCTGTTGCGGGGGAAAAAAGGCAGCATCAGGAAATGGGTGCTGACTGATGGTGCCCCGCCAGAAAGTCACGGCTAACTACGTGCCAGCAGCCG

>LK5::ISJPSFI02F6U7Z AOTU017 454-05

TATAAGAGAGGGTTTGATCCTGGCTCAGGATGAACGCTGGCGGCGTGCCTAACACATGCAAGTCGAACGAAGCATACACCACGGAAGCCTCCGGGCGGAAGAGGGGTATGACTGAGTGGCGGACGGGTGAGTAACACGTGGGCAACCTGCCCTGTACCGGGGGATAACACCCAGAAATGGGTGCTAATACCGCATAAGCGCACAGTGCCGCATGGCACGGTGTGAAAAACTCCGGTGGTACAGGATGGGCCCGCGTCTGATTAGCTAGTTGGCAGGGTAACGGCCTACCAAGGCAGCGATCAGTAGCCGGCTTGAGAGAGTGACCGGCCACATTGGGACTGAGACACGGCCCAAACTCCTACGGGAGGCAGCAGTGGGGAATATTGCACAATGGGGGAAACCCTGATGCAGCGACGCCGCGTGAGTGAAGAAGTATTTCGGTATGTAAAGCTCTATCAGCAGGGAAGAAGATGACGGTACCTGACTAAGAAGCCCCGGCTAACTACGTGCCAGCCGCCGCGGTAAT

>LK5::ISJPSFI02F2W1I AOTU027 454-06

TATAAGAGAGGGTTTGATCCTGGCTCAGGATGAACGCTGGCGGCGTGCTTAACACATGCAAGTCGAACGGAGCACCCTTGAAGGAGTTTTCGGACAACAGATAGGTAATGCTTAGTAGGCGGACGGGTGAGTAACGCGTGAGGAACTTGCCTTGGAGAAGGGGAATAACACAGTGAAAATTGTGCTAATACCGCATGATGCAGTTGGGTCGCATGGCTCTGACTGCCAAAGATTTATCGCTCTGAGATCGGGCCTCGCGTCTGATTAGCTAGTTGGTGGGGCAACGGCCCACCAAGGCGACGATCAGTAGCCGGACTGAGAGGTTGACCGGCCACATTGGGACTGAGACACGGCCCAGACTCCTACGGGAGGCAGCAGTGGGGAATATTGGGCAATGGGCGCAAGCCTGACCCAGCAAACGCCGCGTGAAGGAAGAAGGCTTTCGGGTTGTAAACTTCTTTTAATGAGGGACGAAAGAAATGACGGTACCTCATGAATAAAGCCACGGCTAACTACGTGCCAGCCGCC

>LK5::ISJPSFI02F0VEU AOTU028 454-07

TATAAGAGAGGGTTTGATCATGGCTCAGGATGAACGCTGGCGGCGTGCCTAACACATGCAAGTCGAACGGGTGTACGGGGAGGAAGGCTTCGGCCGGAAAACCTGTGCATGAGTGGCGGACGGGTGAGTAACGCGTGGGCAACCTGGCCTGTACAGGGGGATAACACTTAGAAATAGGTGCTAATACCGCATAACGGGGGAAGCCGCATGGCTTTTCCCTGAAAACTCCGGTGGTACAGGATGGGCCCGCGTCTGATTAGCCAGTTGGCAGGGTAACGGCCCACCAAGGCGACGATCAGTAGCCGGCCTGAGAGGGTGAACGGCCACATTGGGACTGAGACACGGCCCAAACTCCTACGGGAGGCAGCAGTGGGGAATATTGGACAATGGGGGGAACCCTGATCCAGCGACGCCGCGTGAGTGAAGAAGTATTTCGGTATGTAAAGCTCTATCAGCAGGGAAGAAAGAAATGACGGTACCTGACTAAGAAGCCCCGGCTAACTACGTGCCAGCCGCCGCGGTAAT

>LK5::ISJPSFI02F9T7G 5OTU030 454-08

TATAAGAGAGAGTTTGATCCTGGCTCAGGATGAACGCTGGCGGCGTGCCTAACACATGCAAGTCGAACGAAGCATGCAGGAAGAAGCCCTTCGGGGCGGGCGCCTGGATGACTGAGTGGCGGACGGGTGAGTAACGCGTGGGCAACCTGCCCCATACAGGGGGATAACAGCCGGAAACGGCTGCTAATACCGCATAAACCAGGGAGGCGCATGCCTTTCTGGGGAAAGTTCCGGCGGTATGGGAGGGGCCCGCGTCTGATTAGCTTGTTGGCGGGGCAGCGGCCCACCAAGGCGACGATCAGTAGCCGGCCTGAGAGGGCGGACGGCCACATTGGGACTGAGACACGGCCCAAACTCCTACGGGAGGCAGCAGTGGGGGATATTGCACAATGGGGGAAACCCTGATGCAGCGACGCCGCGTGGGCGAAGAAGTATTTCGGTATGTAAAGCCCTATCAGCAGGGAAGAAGAAATGACGGTACCTGAGTAAGAAGCACCGGCTAAATACGTGCCAGCCGCCGCGGTAATT

>LK5::ISJPSFI02F384P 5OTU031 454-09

TATAAGAGAGGGTTTGATCATGGCTCAGGATGAACGCTGGCGGCGTGCCTAACACATGCAAGTCGAACGGACTTAATATGAAACCTAGTGAATGTTAAGTTAGTGGCGGACGGGTGAGTAACGCGTGGATAACCTGCCGTATGCAGGGGGATACCAACTGGAAACAGTTGCTAATACCGCATAAGCGCACAGTGCTGCATGGCACAGTGTGAAAAGAAATTCGGCATACGATGGATCCGCGTCCGATTAGCCAGTTGGCGGGGTAACGGCCCACCAAAGCGACGATCGGTAGCCGGCCTGAGAGGGCGGACGGCCACATTGGGACTGAGACACGGCCCAAACTCCTACGGGAGGCAGCAGTGGGGAATATTGCACAATGGGGGAAACCCTGATGCAGCAACGCCGCGTGAGTGAAGAAGTATTTCGGTACGTAAAGCTCTATCAGCAGGAAAGAAAATGACGGTACCTGACTAAGAAGCCCCGGNTAACTACGTGCCAGCAGCCGCGGTAATT

>LK5::ISJPSFI02F4SC7 5OTU033 454-10

TATAAGAGAGAGTTTGATCATGGCTCAGGATGAACGCTGGCGGCACGCTTAACACATGCAAGTCGAACGAAGCTATAGGGCTTGCCCTATAGACTGAGTGGCAGACGGGTGAGTAACACGTGGGAATTTACCATTTGGTGGGGGATAACATCTGGAAACGGGTGCTAATACCGCATAAGCCCTGAGGGGGAAAGGATTTATTGCCGAATGATAAGCCCGCGCGAGATTAGCTAGTTGGTAAGGTAAGAGCTTACCAAGGCGACGATCTCTAGCTGGTCTGAGAGGATGATCAGCCACACTGGGACTGAGACACGGCCCAGACTCCTACGGGAGGCAGCAGTGGGGAATATTGGACAATGGGGGCAACCCTGATCCAGCGATGCCGCGTGAGTGAAGAAGGCCTTAGGGTTGTAAAGCTCTTTCAGTAGTGAAGATGATGACGGTAACTACAGAAGAAGGACCGGCTAACTCCGTGCCAGCAGCCGCGGTAATT

>LK5::ISJPSFI02F2NP1 AOTU035 454-11 (179)

TATAAGAGAGGGTTTGATCATGGCTCAGGATGAACGCTGGCGGCGTGCCTAACACATGCAAGTCGAACGGGTGTACAGAAGGGAAGATTACGGTCGGAAGATCTGTGCATGAGTGGCGGACGGGTGAGTAACGCGTGGGCAACCTGGCCTGTACAGGGGGATAACACTTAGAAATAGGTGCTAATACCGCATAACGGGGGAAGCCGCATGGCTTTTTCCTGAAAACTCCGGTGGTACAGGATGGGCCCGCGTCTGATTAGCTGGTTGGCAGGGTAACGGCCTACCAAGGCGACGATCAGTAGCCGGCCTGAGAGGGCGGACGGCCACACTGGGACTGAGACACGGCCCAGACTCCTACGGGAGGCAGCAGTGGGGGATATTGCACAATGGGGGGAACCCTGATGCAGCGACGCCGCGTGGGTGAAGGAGCGTTTCGGCGCGTAAAGCCCTATCGGCAGGGAAGAAGGAGGACGGTACCTGACTAAAGAAGCCCCGGCTAACTACGTGCCAGCCGCCGCGGTAAT

>LK5::ISJPSFI02G0P2Q 5OTU039 454-12

TATAAGAGAGAGTTTGATCATGGCTCAGGATGAACGCTGGCGGCGTGCCTAATACATGCAAGTCGAGCGAACCACTTCGGTGGGAAGCGGCGAACGGGTGAGTAACACGTAGGTGATCTGCCCATCAGACGGGGACAACGATTGGAAACGATCGCTAATACCGGATAGGACGAAAGTTTAAAGATGCTCCTGGCATCACTGATGGATGAGCCTGCGGCGCATTAGCTAGTTGGTGGGGTAAAGGCCTACCAAGGCGACGATGCGTAGCCGACCTGAGAGGGTGAACGGCCACACTGGGACTGAGACACGGCCCAGACTCCTACGGGAGGCAGCAGTAGGGAATCTTCGGCAATGGGCGAAAGCCTGACCGAGCAACGCCGCGTGAATGAAGAAGGCCTTCGGGTTGTAAAATTCTGTTATAAGGGAAGAAAGGTGATAGGAGGAAATGACTATCAATTGACGGTACCTTATGAGAAAGCCACGGCTAACTACGTGCCAGCCGCCGCGGTAAT

>LK5::ISJPSFI02H82CT 5OTU040 454-13

TATAAGAGAGGGTTTGATCATGGGCTCAGGACGAACGCTGGCGGCGTTGCCTAACACATGCAAGTCGAACGAAGCTTGACGAATGATTTCTTCGGAATGAAATCTGATATGACTGAGTGGCGGACGGGTGAGTAACGCGTGAGCAACCTGCCCTTCGGAGGGGGATAGTGTCTGGAAACGGACAGTAATACCGCATAACGTATTTTTACCGCATGATAGAAATACCAAAACTGAGGTGCCGAAGGATGGGCTCGCGTTGGATTAGATAGTTGGTGGGGTAACGGCCTACCAAGTCGACGATCCATAGCCGGACTGAGAGGTTGAACGGCCACATTGGGACTGAGACACGGCCCAGACTCCTACGGGAGGCAGCAGTGGGGGATATTGCACAATGGGGGAAACCCTGATGCAGCGACGCCGCGTGGGCGAAGAAGTATTTCGGTATGTAAAGCCCTATCAGCAGGGAANAAGAAATGGCGGTACCTGAGTAAGAAGCACCGGCTAAATACGTGCCAGCAGCCGCGGTAATT

>LK5::ISJPSFI02GXC07 AOTU043 454-14

TATAAGAGAGGGTTTGATCCTGGCTCAGGATGAACGCTGGCGGCGTGCCTAACACATGCAAGTCGAACGGGTGTACGGGGAGGAAGGCTTCGGCCGGAAAACCTGTGCATGAGTGGCGGACGGGTGAGTAACGCGTGGGCAACCTGGCCTGTACAGGGGGATAACACTTAGAAATAGGTGCTAATACCGCATAACGGGGGAAGCCGCATGGCTTTTCCCTGAAAACTCCGGTGGTACAGGATGGGCCCGCGTCTGATTAGCCAGTTGGCAGGGTAACGGCCTACCAAAGCGACGATCAGTAGCCGGCCTGAGAGGGCGGACGGCCACATTGGGACTGAGACACGGCCCAAACTCCTACGGGAGGCAGCAGTGGGGAATATTGCACAATGGGGGAAACCCTGATGCAGCAACGCCGCGTGAAGGAAGACGGTTTTCGGATTGTAAACTTCTTTTCTTAGTGAAGAAGCAAGTGACGGTAGCTAAGGATAAGCATCGGCTAACTACGTGCCAGCCGCCGCGGTAATT

>LK5::ISJPSFI02F4W2D 5OTU051 454-15

TATAAGAGAGAGTTTGATCCTGGCTCAGGATGAACGCTGGCGGCGTGCCTAACACATGCAAGTCGAACGAAGTTACTTGCTTAGATCTCTTCGGAGTGACGAGCTTTTAACTTAGTGGCGGACGGGTGAGTAACACGTGAGCAACCTGCCTTTCAGAGGGGGATAACGGCTGGAAACGGTCGCTAATACCGCATGATATATTGAATCGGCATCGGTTTGATATCAAAGGAGCAATCCGCTGAAAGATGGGCTCGCGTCTGATTAGATAGTTGGTGGGTAACGGCCTACCAAGTCGACGATCAGTAGCCGGACTGAGAGGTTGAACGGCCACATTGGGACTGAGATACGGCCCAGACTCCTACGGGAGGCAGCAGTGGGGAATATTGCACAATGGGGGAAACCCTGATGCAGCAACGCCGCGTGAAGGAAGACGGTTTTCGGATTGTAAACTTCTTTTCTCAGGGACGAAGCAAGTGACGGTACCTGAGGAATAAGCCACGGCTAACTACGTGCCAGCAGCCGCGGTAA

>LK5::ISJPSFI02G1JB3 5OTU053 454-16

TATAAGAGAGGGTTTGATCATGGCTCAGGATGAACGCTGGCGGCGTGCCTAACACATGCAAGTCGAACGGGCATGCGCATGAAACCTAGTGATTGCGCATGCGAGTGGCGGACGGGTGAGTAACGCGTGGACAACCTGCCGCATGCAGGGGGATACCGGCTGGAAACAGCCGCTAATACCGCATATGCGCACGGCGCCGCATGGCGCAGTGCGGAAAGGGAGCGATTCCGGCATGCGATGGGTCCGCGTCCGATTAGCTTGTTGGCGGGGCAGCGGCCCACCAAGGCGACGATCGGTAGCCGGCCTGAGAGGGCGGACGGCCACATTGGGACTGAGACACGGCCCAGACTCCTACGGGAGGCAGCAGTGGGGGATATTGCACAATGGGGGAACCCTGATGCAGCGACGCCGCGTGGGTGAAGGAGTGCTTCGGCATGTAAAGCCCTATCGGCAGGGAAGAAGCAAGACGGTACCTGA

>LK5::ISJPSFI02GK9VE56 AOTU056 454-17

TATAAGAGAGGGTTTGATCCTGGCTCAGGATGAACGCTGGCGGCGTGCCTAACACATGCAAGTCGAACGGACTTAACCTGAAACCTAGTGATTGTTAAGTTAGTGGCGGACGGGTGAGTAACGCGTGGATAACCTGCCGTATGCAGGGGGATAACAGTTGGAAACAGCTGCTAATACCGCATAAGCGCACAGTACCGCATGGTGCAGTGCGAAAAGATTTATCGGCATACGATGGATCCGCGTCCGATTAGCCAGTTGGCGGGGTAACGGCCCACCAAAGCGACGATCGGTAGCCGGCCTGAGAGGGCGGACGGCCACATTGGGACTGAGACACGGCCCAAACTCCTACGGGAGGCAGCAGTGGGGAATATTGCACAATGGGGGAAACCCTGATGCAGCGACGCCGCGTGGGTGAAGAAGTACTTCGGTACGTAAAGCCCTATCAGCAGGGGAAGAAGGAGGACGGTACCTGACTAAGAAGCCCCCGGCTAACTACGTGCCAGCAGCCGCGGTAATT

>LK5::ISJPSFI02FONUD 5OTU061 454-18

TATAAGAGAGAGTTTGATCATGGCTCAGGATGAACGCTGGCGGCGTGCCTAACACATGCAAGTCGAACGGGTGCGTGCAGGAAGGGCGGGGTCAAACCAGCCCGGAGGGTGCGCATGAGTGGCGGACGGGTGAGTAACGCGTGGGCAACCTGCCGTATACAGGGGGATAACACCCGGAAACGGGTGCTAATACCGCATAAGCGCACGAGTGCCGCATGGCACGGTGTGAAAAACTCCGGTGGTATACGATGGGCCCGCGTCCGATTAGCTTGTTGGCGGGGCAGCGGCCCACCAAGGCGACGATCGGTAGCCGGCCTGAGAGGGCGGACGGCCACATTGGGACTGAGACACGGCCCAAACTCCTACGGGAGGCAGCAGTGGGGGATATTGCACAATGGGGGGAACCCTGATGCAGCGACGCCGCGTGAGTGAAGAAGCGTCTCGGCGCGTAAAGCTCTATCGGCAGGGAAGAGGGGGGGCGACCCCGGACGGTACCTGACTAAGAAGCCCCGGCTAACTACGTGCCAGCAGCCGCGGTAAT

>LK5::ISJPSFI02FQQF0 5OTU062 454-19

TATAAGAGAGAGTTTGATCCTGGCTCAGGACGAACGCTGGCGGCGTGCCTAACACATGCAAGTCGAACGGAGTTACGATGAAACCTAGTGATTCGTAACTTAGTGGCGGACGGGTGAGTAACGCGTGGATAACCTGCCTTGCACTGGGGGATAACACTTAGAAATAGGTGCTAATACCGCATAAGCGCACAGAGCCGCATGGCTCAGTGTGAAAAACTCCGGTGGTGTAAGATGGATCCGCGTCTGATTAGCTGGTTGGCGGGGTAGAAGCCCACCAAGGCGACGATCAGTAGCCGGCCTGAGAGGGTGAACGGCCACATTGGGACTGAGACACGGCCCAAACTCCTACGGGAGGCAGCAGTGGGGAATATTGCACAATGGGGGAAACCCTGATGCAGCAACGCCGCGTGAGTGAAGAAGTCATTCGTGATGTAAAGCTCTATCAGCAGGGAAGAAAATGACGGTACCTGAGTAAGAAGCCCCGGCTAACTACGTGCCAGCAGCCGCGGTAAT

>LK5::ISJPSFI02F1DGK 5OTU063 454-20

TATAAGAGAGAGTTTGATCATGGCTCAGGATGAACGCTGGCGGCGTGCCTAATACATGCAAGTCGAACGCGGGCACTTGTGCCCGAGTGGCGAACGGGTGAGTAATACATAAGTAACCTGGCCTTTACAGGGGGATAACTGCTGGAAACGGCAGCTAAGACCGCATAGGTAGGGACACTGCATGGTGACCGTATTAAAAGTGCTGCAAGGCACTAGTAGAGGATGGACTTATGGCGCATTAGCTGGTTGGTGAGGTAACGGCTCACCAAGGCGACGATGCGTAGCCGACCTGAGAGGGTGACCGGCCACACTGGGACTGAGACACGGCCCAGACTCCTACGGGAGGCAGCAGTAGGGAATTTTCGGCAATGGGGGGGAACCCTGACCGAGCAACGCCGCGTGAAGGAAGAAGGAATTCGTTCTGTAAACTTCTGTTATAAAGGAAGAAAGACGGATGGAGGAAATGACATCCGAGTGACGGTACTTTATGAGAAAGCCACGGCTAACTACGTGCCAGCCGCCGCGGTAAT

>LK5::ISJPSFI02FT2L0 5OTU064 454-21

TATAAGAGAGGGTTTGATCCTGGCTCAGGATGAACGCTGGCGGCGTGCCTAACACATGCAAGTCGAACGGGTGCGTGCAGGAAGGGCGGGGTCAAACCAGCCCGGAGGGTGCGCATGAGTGGCGGACGGGTGAGTAACGCGTGGGCAACCTGCCGTATACAGGGGGATAACACCCGGAAACGGGTGCTAATACCGCATAAGCGCACAGTGCCGCATGGCACGGTGTGAAAAACTCCGGTGGTACAGGATGGGCCCGCGTCTGATTAGCTGGTTGGCAGGGTAACGGCCTACCAAGGCGACGATCAGTAGCCGGCCTGAGAGGGCGGACGGCCACACTGGGACTGAGACACGGCCCAGACTCCTACGGGAGGCAGCAGTGGGGGATATTGCACAATGGGGGGAACCCTGATGCAGCGACGCCGCGTGGGTGAAGAAGCGCCTCGGCGCGTAAAGCCCTGTCAGCAGGGAAGAAAATGACGGTACCTGAAGAAGAAGCCCCGGCTAACTACGTGCCAGCAGCCGCGGTAAT

>LK5::ISJPSFI02FRWS7 5OTU067 454-22

TATAAGAGAGGGTTTGATCCTGGCTCAGGATGAACGCTGGCGGCGTGCCTAACACATGCAAGTCGAACGGACTTAACCTGAAACCTAGTGATAGTTAAGTTAGTGGCGGACGGGTGAGTAACGCGTGGATAACCTGCCGTATGCAGGGGGATACCAACTGGAAACAGTTGCTAATACCGCATAAGCGCACGGTACCGCATGGTACAGTGTGAAAAGAATTATCGGCATACGATGGACCCGCGTCCGATTAGCCAGCTGGCGGGGTAAAGGCCCACCAAAGCGACGATCGGTAGCCGGCCTGAGAGGGCGGACGGCCACACTGGGACTGAGACACGGCCCAGACTCCTACGGGAGGCAGCAGTGGGGAATATTGCACAATGGGGGGAACCCTGATGCAGCGACGCCGCGTGGGTGAAGGAGTATTTCGGTATGTAAAGCCCTATCAGCAGGGAAGAACAATGACGGTACCTGACTAAGAAGCACCGGCTAAATACGTGCCAG

>LK5::ISJPSFI02G0CSI 5OTU075 454-23

TATAAGAGAGAGTTTGATCCTGGCTCAGGACGAACGCTGGCGGCATGCCTAACACATGCAAGTCGAACGGAGTTATTATTCAGAAGTTCTTCGGAATGGAAGGATAAGAACTTAGTGGCGGACGGGTGAGTAACGCGTGGGTAACCTGCCCTTTTGTGGGGAACAACTTCGAGAAATCGGAGCTAATACCGCATAATAATAGAGGATCGCATGATTCTTTAAGGAAAGATGGCCTCTGAAGATGCTATCGCAAAAGGATGGACCCGCGTCTGATTAGCTAGTTGGAAGGGTAACGGCCTACCAAGGCAACGATCAGTAGCCGGCCTGAGAGGGTGAACGGCCACATTGGGACTGAGACACGGCCCAAACTCCTACGGGAGGCAGCAGTGGGGAATATTGCACAATGGGGGAAACCCTGATGCAGCGACGCCGCGTGGGTGAAGAAGCGCCTCGGCGCGTAAAGCCCTGTCAGCAGGGAGAAAATGACGGTACCTGAAGAAGAAGCCCCGGCTAAC

>LK5::ISJPSFI02F3USB AOTU079 454-24

TATAAGAGAGAGTTTGATCATGGCTCAGGATGAACGCTGGCGGCGTGCCTAACACATGCAAGTCGAACGGGTGTACAGAAGGGAAGATTACGGTCGGAAGATCTGTGCATGAGTGGCGGACGGGTGAGTAACGCGTGGGCAACCTGGCCTGTACAGGGGGATAACACTTAGAAATAGGTGCTAATACCGCATAACGGGAGAAGCCGCATGGCTTTTTCCTGAAAACTCCGGTGGTACAGGATGGGCCCGCGTCTGATTAGCTAGTTGGCAGGGTAACGGCCTACCAGGGCAGCGATCAGTAGCCGGCTTGAGAGAGTGACCGGCCACATTGGGACTGAGACACGGCCCAAACTCCTACGGGAGGCAGCAGTGGGGAATATTGCACAATGGGGGAAACCCTGACGCAGCGACGCCGCGTGAGTGAAGAAGTATTTCGGTATGTAAAGCTCTATCAGCAGGGAAGAAGATGACGGTACCTGACTAAGAAGCCCCGGNTAACTACGTGCCAGCAGCCGCGGTAAT

>LK5::ISJPSFI02G3PLF 5OTU098 454-25

TATAAGAGAGAGTTTGATCCTGGCTCAGGATGAACGCTGGCGGCGTGCTTAACACATGCAAGTCGAACGAAGCACGGGGACGGAAGCCCTCCGGGGTGGAAGGCCGCGTGACTGAGTGGCGGACGGGTGAGTAGCGCGTGGGTAACCTGCCCCATACAGGGGGATAACAGCCGGAAACGGCTGCTAATACCGCATGCACATGCGGGGGCGCATGCCCCTGCATGGAAAGCTTTCGCGGTATGGGATGGGCCCGCGTCTGATTAGGCAGTTGGCGGGGTAACGGCCCACCAAACCGACGATCAGTAGCCGGCCTGAGAGGGCAACCGGCCACATTGGGACTGAGACACGGCCCAAACTCCTACGGGAGGCAGCAGTGGGGAATATTGCACAATGGGGGGAACCCTGATGCAGCGACGCCGCGTGGGTGAAGGAGTATTTCGGTATGTAAAGCCCTATCAGCAGGGAAGAACAATGACGGTACCTGACTAAGAAGCACCGGCTAAATACGTGCCAGCAGCCGCGGTAAT

>LK5::ISJPSFI02F721S AOTU117 454-26

TATAAGAGAGAGTTTGATCCTGGCTCAGGATGAACGCTGGCGGCGTGCCTAACACATGCAAGTCGAACGGGTGTACGGGGAGGAAGGCTTCGGCCGGAAAACCTGTGCATGAGTGGCGGACGGGTGAGTAACGCGTGGGCAACCTGGCCTGTACAGGGGGATAACACTTAGAAATAGGTGCTAATACCGCATAACGGGGGAAGCCGCATGGCTTTTCCCTGAAAACTCCGGTGGTACAGGATGGGCCCGCGTCTGATTAGCCAGTTGGCAGGGTAACGGCCTACCAAAGCGACGATCAGTAGCCGGCCTGAGAGGGCGGACGGCCACATTGGGACTGAGACACGGCCCAAACTCCTACGGGAGGCAGCAGTGGGGAATATTGCACAATGGGGGAAACCCTGATGCAGCGACGCCGCGTGGGCGAGGGAGCGCCTCGGCGCGTAAAGCCCTTTCGGCGGGGGAGAACGATGACGGTACCCGACGAAGAAGCACCGGCTAAATACGTGCCAGCCGCCGCGGTAATT

>LK5::ISJPSFI02HIRQK 5OTU121 454-27

TATAAGAGAGAGTTTGATCCTGGCTCAGGATGAACGCTGGCGGCGTGCCTAACACATGCAAGTCGAGCGATTTACTTCGGTAAAGAGCGGCGGACGGATGAGTAACGCGTGGGTAACCTGCCCTGTACACACGGATAACATACCGAAAGGTATGCTAATACGAGATAAAATACTTTTATCGCATGGTAGAAGTATCAAAGCTTTTGCGGTACAGGATGGACCCGCGTCTGATTAGCTAGTTGGTAAGGTAACGGCTTACCAAGGCGGCGATCAGTAGCCGACCTGAGAGGGTGATCGGCCACATTGGAACTGAGACACGGTCCAAACTCCTACGGGAGGCAGCAGTGGGGAATATTGCACAATGGGCGAAAGCCTGATGCAGCAACGCCGCGTGAGCGATGAAAGGCCTTCGGGTCGTAAAGCTCTGTCCTCAAGGAAGATAATGACGGTACTTGAGGAGGAAGCCCCGGCTAACTACGTGCCAGCCGCCGCGGTAATT

>LK5::ISJPSFI02FRYER 5OTU137 454-28

TATAAGAGAGGGTTTGATCATGGCTCAGGATGAACGCTTGCGGCGTGCCTAACACATGCAAGTCGAACGAAGCATGCAGGAAGAAGCCCTTCGGGGCGGGCGCCTGGATGACTGAGTGGCGGACGGGTGAGTAACGCGTGGGCAACCTGCCCCATACAGGGGGATAACAGCCGGAAACGGCTGCTAATACCGCATAAACCAGGGAGGCGCATGCCTTTGTGGGGAAAGCTCCGGCGGTATGGGAGGGGCCCGCGTCTGATTAGCTTGTTGGCGGGGCAGCGGCCCACCAAGGCGACGATCAGTAGCCGGCCTGAGAGGGCGACGGCCACATTGGGACTGAGACACGGCCCAAACTCCTACGGGAGGCAGCAGTGGGGGATATTGCACAATGGGGGAAACCCTGATGCAGCGACGCCGCGTGGGCGAAGAAGTATTTCGGTATGTAAAGCCCTATCAGCAGGGAAGAAGGAGGACGGTACCTGACTAAGAAGCCCCGGCTAACTACGTGCCAGCAGCCGCGGTAATT

>LK5::ISJPSFI02F1QOD 5OTU144 454-29

TATAAGAGAGGGTTTGATCCTGGCTCAGGATGAACGCTGGCGGCGTGCTTAACACATGCAAGTCGAGCGAAGCACTTTGGTTAGACTCTTCGGATGAAGACTTTAGTGACTGAGCGGCGGACGGGTGAGTAACGCGTGGGTAACCTGCCTCATACAGGGGGATAACAGTTAGAAATGACTGCTAATACCGCATAAGACCACAGAGCCGCATGGCCCGGTGGTAAAAACTCCGGTGGTATGAGATGGACCCGCGTCTGATTAGCTGGTTGGTGGGGTAACGGCCTACCAAGGCGACGATCAGTAGCCGACCTGAGAGGGTGACCGGCCACATTGGGACTGAGACACGGGCCCAAACTCCTACGGGAGGCAGCAGTGGGGAATATTGCACAATGGGGCAAAAGCCTGATGCAGCGACGCCGCGTGAAGGATGAAGTA

>LK5::ISJPSFI02G0KIZ 5OTU162 454-30

TATAAGAGAGGGTTTGATCATGGCTCAGGATGAACGCTGGCGGCGTGCCTAACACATGCAAGTCGAACGGGTGTACGGGATGGAAGATTTCGGTCGGAAGCCCCGTGCATGAGTGGCGGACGGGTGAGTAACGCGTGGGCAACCTGCCCTGTACAGGGGGACAACACTTAGAAATAGGTGCTAATACCGCATAACGGGGGGAGCCGCATGGCTTCCCCCTGAAAACTCCGGTGGTACAGGATGGGCCCGCGTCTGATTAGCCAGTTGGCAGGGTAACGGCCTACCAAAGCGACGATCAGTAGCCGGCCTGAGAGGGCGGACGGCCACACTGGGACTGAGACACGGCCCAGACTCCTACGGGAGGCAGCAGTGGGGGATATTGCACAATGGGGGGAACCCTGATGCAGCGACGCCGCGTGAGTGAAGAAGCGTCTCGGCGCGTAAAGCTCTATCGGCAGGGAAGAGGGGGGCGACCCCGGGACGGTACCTGACTAAGAAGCCCCGGCTAACTACGTGCCAGCAGCCGCGGTAAT

>LK5::ISJPSFI02JZ3S6 5OTU179 454-31

TATAAGAGAGGGTTTGATCCTGGCTCAGGATGAACGCTGGCGGCGTGCCTAACACATGCAAGTCGAACGGGCGTACGGGATGGAAGGCTCCGGCCGGAAGCCCCGTGCATGAGTGGCGGACGGGTGAGTAACGCGTGGGCAACCTGCCCTGTACAGGGGGACAACACTTAGAAATAGGTGCTAATACCGCATAACGGGGGGAGCCGCATGGCACCCCCCTGAAACTACCGGTGGTATAGGAGTGGGCCCGGCCGTCTGATTAGCTGGTTGGCAGGGTAACGGGCCTACCAAGGCGACGATCAGTAGCCGGCCTGAGAGGGCGGACGGCCACACTGGGACGTGAGACACGGCCCACGACTCCTACGGGAGGGCAGCAGTGGGGGAGTATTGCACAATGGGGGGAACCCGTGATGCAGCGACGCCGCGTGGGTGAAGGAGCGTTTCGGCGCGTAAAGCCCTATCGGCAGGGAAGAAGGAGGACGGTACCTGACTAAGAAGCCCCGGCTAACTACGTGCCAGCCGCCGCGGTAAT>LK5::ISJPSFI02FSYQ0 5OTU185 454-32

TATAAGAGAGGGTTTGATCATGGCTCAGGATGAACGCTGGCGGCGTGCCTAACACATGCAAGTCGAACGGACTTAATATGAAACCTAGTGAATATTAAGTTAGTGGCGGACGGGTGAGTAACGCGTGGATAACCTGCCGTATGCAGGGGGATACCAACTGGAAACAGTTGCTAATACCGCATAAGCGCACAGTGCTGCATGGCACAGTGTGAAAAGAAATTCGGCATACGATGGATCCGCGTCCGATTAGCCAGTTGGCGGGGTAACGGCCTACCAAGGCGACGATCAGTAGCCGGCCTGAGAGGGCGGACGGCCACACTGGGACTGAGACACGGCCCAGACTCCTACGGGAGGCAGCAGTGGGGGATATTGCACAATGGGGGGAACCCTGATGCAGCGACGCCGCGTGGGTGAAGAAGCGCCTCGGCGCGTAAAGCCCTGTCAGCAGGGAAGAAAATGACGGTACCTGACTAAGAAGCCCCGGCTAACTACGTGCCAGCCGCCGCGGTAATT

>LK5::ISJPSFI02H90ON 5OTU189 454-33

TATAAGAGAGGGTTTGATCCTGGCTCAGGATGAACGCTGGCGGCGTGCCTAACACATGCAAGTCGAACGAAGCATACACCACGGAAGCCTCCGGGCGGAAGAGGGGTATGACTGAGTGGCGGACGGGTGAGTAACGCGTGGGCAACCTGCCCTGTACCGGGGGATAACACCCAGAAATGGGTGCTAATACCGCATAAGCGCACAGTGCCGCATGGCACGGTGTGAAAAACTCCGGTGGTACAGGATGGGCCCGCGTCTGATTAGCTAGTTGGCAGGGTAACGGCCTACCAAGGCAGCGATCAGTAGCCGGCTTGAGAGAGTGACCGGGCCACATTGGGACTGAGACACGGCCCCAGACTCCTACGGGAGGCAGCAGTGGGGGATATTGCACAATGGGGGGAACCCTGATGCAGCGACGCCGCGTGGGTGAAGAAGCGCCTCGGCGCGTAAAGCCCCTGTCAGCAGGGAAGAAAATGACGGTACCTGAAGAAGAAGCCCCGGCTAACTACGTGCCAGCAGCCGCGGTAATT

>LK5::ISJPSFI02F1FKN 5OTU205 454-34

TATAAGAGAGGGTTTGATCCTGGCTCAGGACGAACGCTGGCGGCATGCCTAACACATGCAAGTCGAACGGAGTTATTATTCAGAAGTTCTTCGGGATGGAAGGATAAGTAACTTAGTGGCGGACGGGTGTAGTAACGCGTGGGTAACCTGCCCTTTTGTGGGGAACAACTTCGAGAAATCGGAGCTAATACCGCATAATAATAGAGGATCGCATGATTCTTTAAGGAAAGATGGCCTCTGAAGATGCTATCGCAAAAAGGATGGACCCGCGTCTGATTAGCTAGTTGGAAGGGTAACGGCCTACCAAGGCAATGATCAGTAGCCGGCCTGAGAGGGTGAACGGCCACATTGGGACTGAGACACGGCCCAGACTCCTGCGGGAGGCAGCAGTGGGGAATATTGCACAATGGGGGAAACCCTGATGCAGCGACGCCGCGTGAGCGAAGAAGTATTTCGGTATGTAAAAGCTCTATCAGCAGGGAAGAAAATGACGGTACCTGAGTAAGAAGCTCCGGCTAAATACGTGCCGGCCGCC

>LK5::ISJPSFI02F8F7O 5OTU319 454-35

TATAAGAGAGAGTTTGATCCTGGCTCAGGATGAACGCTGGCGGCGTGCCTAACACATGCAAGTCGAACGGGTGTACGGGGAGGAAGGCTTCGGCCGGAAAACCTGTGCATGAGTGGCGGACGGGTGAGTAACGCGTGGGCAACCTGCCCTGTACAGGGGGACAACACTTAGAAATAGGTGCTAATACCGCATAACGGGGGGAGCCGCATGGCTTCCCCCTGAAAACTCCGGTGGTACAGGATGGGCCCGCGTCTGATTAGCTGGTTGGCAGGGTAACGGCCTACCAAGGCGACGATCAGTAGCCGGCCTGAGAGGGCGGACGGCCACACTGGGACTGAGACACGGCCCAGACTCCTACGGGAGGCAGCAGTGGGGGATATTGCACAATGGGGGGAACCCTGATGCAGCGACGCCGCGTGGGTGAAGGAGCGTTTCGGCGCGTAAAGCCCTATCGGCAGGGAAGAAGGAGGACGGTACCTGACTAAGAAGCCCCGGCTAACTACGTGCCAGCAGCCGCGCGGTAATT

>LK5::ISJPSFI02G78E2 5OTU411 454-36

TATAAGAGAGGGTTTGATCCTGGCTCAGGATGAACGCTGGCGGCGTGCCTAACACATGCAAGTCGAACGAAGTTACTTGCTTAGATCTCTTCGGAGTGACGAGCTTTTAACTTAGTGGCGGACGGGTGAGTAACACGTGAGCAACCTGCCTTTCAGAGGGGGATAACGGCTGGAAACGGTCGCTAATACCGCATGATATATTGAATCGGCATCGGTTTGATATCAAAGGAGCAATCCGCTGAAAGATGGGCTCGCGTCTGATTAGATAGTTGGTGGGGTAACGGCCTACCAAAGCGACGATCAGTAGCCGGCCTGAGAGGGCGGACGGCCACACTGGGACTGAGACACGGCCCAGACTCCTACGGGAGGCAGCAGTGGGGGATATTGCACAATGGGGGGAACCCTGATGCAGCGACGCCGCGTGGGTGAAGAAGCGCCTCGGCGCGTAAAGCCCTGTCAGCAGGGAGAAAATGACGGTACCTGAAGAAGAAGCCCCGGCTAACTACGTGCCAGCAGCCGCGGTAA

>LK5::ISJPSFI02FPL5N 5OTU446 454-37

TATAAGAGAGGGTTTGATCATGGCTCAGGATGAACGCTGGCGGCGTGCCTAACACATGCAAGTCGAACGAAGCATGCAGGAAGAAGCCCTTCGGGGCGGGCGCCTGGATGACTGAGTGGCGGACGGGTGAGTAACGCGTGGGCAACCTGCCCCATACAGGGGGATAACAGCCGGAAACGGCTGCTAATACCGCATAAACCAGGGAGGCGCATGCCTTTGTGGGGAAAGCTCCGGCGGTATGGGAGGGGCCCGCGTCTGATTAGCTTGTTGGCGGGGCAGCGGCCCACCAAGGCGACGATCAGTAGCCGGCCTGAGAGGGCGGACGGCCACATTGGGACTGAGACACGGCCCAAACTCCTACGGGAGGCAGCAGTGGGGAATATTGCACAATGGGGGAAACCCTGATGCAGCAACGCCGCGTGAGTGAAGAAGTATTTCGGTACGTAAAGCTCTATCAGCAGGAAAGAAAATGACGGTACCTGACTAAGAAGCCCCGGCTAACTACGTGCCAGCAGCCGCGGTAATT

>LK5::ISJPSFI02FMPFL 5OTU535 454-38

TATAAGAGAGGGTTTGATCATGGCTCAGGATGAACGCTGGCGGCGTGCCTAACACATGCAAGTCGAACGGGTGTACGGGGAGGAAGGCTTCGGCCGGAAAACCTGTGCATGAGTGGCGGACGGGTGAGTAACGCGTGGGCAACCTGGCCTGTACAGGGGGATAACACTTAGAAATAGGTGCTAATACCGCATAACGGGGGAAGCCGCATGGCTTTTCCCTGAAAACTCCGGTGGTACAGGATGGGCCCGCGTCTGATTAGCCAGTTGGCAGGGTAACGGCCTACCAAAGCGACGATCAGTAGCCGGCCTGAGAGGGCGGACGGCCACACTGGGACTGAGACACGGCCCAGACTCCTACGGGAGGCAGCAGTGGGGGATATTGCACAATGGGGGGGAACCCTGATNGC

>LK5::ISJPSFI02F5ELZ 5OTU541 454-39

TATAAGAGAGAGTTTGATCCTGGCTCAGGATGAACGCTGGCGGCGTGCCTAACACATGCAAGTCGAACGGGTGTACGGGGAGGAAGGCTTCGGCCGGAAAACCTGTGCATGAGTGGCGGACGGGTGAGTAACGCGTGGGCAACCTGGCCTGTACAGGGGGATAACACTTAGAAATAGGTGCTAATACCGCATAACGGGGGAAGCCGCATGGCTTTTCCCTGAAAACTCCGGTGGTACAGGATGGGCCCGCGTCTGATTAGCTGGTTGGCAGGGTAACGGCCTACCAAGGCGACGATCAGTAGCCGGCCTGAGAGGGCGGACGGCCACATTGGGACTGAGACACGGCCCAGACTCCTACGGGAGGCAGCAGTGGGGAATATTGCACAATGGGGGAAACCCTGATGCAGCGACGCCGCGTGAGCGATGAAGTATTTCGGTATGTAGAGCCCTATCGGCAGGGAAGAAGAAATGACGGTACCTGAATAAGAAGCACCGGCTAAATACGTGCCAGCAGCCGCGGTAAT

>LK5::ISJPSFI02F59LG 5OTU614 454-40

TATAAGAGAGGGTTTGATCCTGGCTCAGGATGAACGCTGGCGGCGTGCCTAACACATGCAAGTCGAACGGGTGTACGGGGAGGAAGGCTTCGGCCGGAAAACCTGTGCATGAGTGGCGGACGGGTGAGTAACGCGTGGGCAACCTGGCCTGTACAGGGGGATAACACTTAGAAATAGGTGCTAATACCGCATAACGGGGGAAGCCGCATGGCTTTTCCCTGAAAACTCCGGTGGTACAGGATGGGCCCGCGTCTGATTAGCTGGTTGGCAGGGTAACGGCCTACCAAGGCGACGATCAGTAGCCGGCCTGAGAGGGTGACCGGCCACATTGGGACTGAGACACGGCCCAAACTCCTACGGGAGGCAGCAGTGGGGAATATTGCACAATGGGGGAAACCCTGATGCAGCGACGCCGCGTGAGTGAAGAAGTATTTCGGTATGTAAAGCTCTATCAGCAGGGAAGAAAACGACGGTACCTGACTAAGAAGCCCCGGCTAACTACGTGCCAGCAGCCGCGGTAATT

>LK5::ISJPSFI02GNEG0 5OTU624 454-41

TATAAGAGAGGGTTTGATCATGGCTCAGGATGAACGCTGGCGGCGTGCCTAACACATGCAAGTCGAACGGGTGTACAGAAGGGAAGATTACGGTCGGAAGATCTGTGCATGAGTGGCGGACGGGTGAGTAACGCGTGGGCAACCTGGCCTGTACAGGGGGATAACACTTAGAAATAGGTGCTAATACCGCATAACGGGAGAAGCCGCATGGCTTTTTCCTGAAAACTCCGGTGGTACAGGATGGGCCCGCGTCTGATTAGCCAGTTGGCAGGGTAACGGCCTACCAAAGCGACGATCAGTAGCCGGCCTGAGAGGGCGGACGGCCACACTGGGACTGAGACACGGCCCAAACTCCTACGGGAGGCAGCAGTAGGGAATCTTCCGCAATGGGCGAAAGCCTGACGGAGCAATGCCGCGTGAGTGAAGAAGGCCTTCGGGTTGTAAAACTCTGTCCTTATCGAAGAGAGGTAGGTATGTGAATAATGTACCTATAGGACGGTAGATAAGGAGGAAGCCCCGGCTAACTACGTGCCAACAGCCGC

>LK5::ISJPSFI02JL7W2 5OTU633 454-42

TATAAGAGAGGGTTTGATCCTGGCTCAGGATGAACGCTGGCGGCGTGCCTAACACATGCAAGTCGAACGGGTGTACGGGATGGAAGATTTCGGTCGGAAGCCCCGTGCATGAGTGGCGGACGGGTGAGTAACGCGTGGGCAACCTGCCCTGTACAGGGGGACAACACTTAGAAATAGGTGCTAATACCGCATAACGGGGGGAGCCGCATGGCACCCCCCTGTAAACTACCGGTGGTACAGGATGGGCCCGGCGTCTGATTAGCTGGTTAGGTCAGGGTAACGGGCCTACCAAGGCGACCGATCAGTAGCCGGCCTGAGAGGGCGGACGGCCACACTGGGACGTGAGGACACGGCCCAGAACTCCTACGGGAGGCAGCAGTGGGGGATATTGCACAATGGGGGAACCCGTGATGCAGCGACGCCGCGTGGGTGAAGGAGCGTTTCGGCGCGTAAAGCCCTATCGGCAGGGAAGAAGGAGGAC

>LK5::ISJPSFI02HO2ZS 5OTU642 454-43

TATAAGAGAGGGTTTGATCCTGGCTCAGGATGAACGCTGGCGGCGTGCCTAACACATGCAAGTCGAACGGGTGTACGGGATGGAAGATTTCGGTCGGAAGCCCCGTGCATGAGTGGCGGACGGGTGAGTAACGCGTGGGCGAACCTGCCCTGTACAGGGGGACGAACACTTAGAAATAGGTGCTAATACCGCATAACGGGGGAGCCGCATGGCTTCCCCCTCGAAAACTACCGGTGGTACAGGATGGGCCCGCGTCTGATTAGCTGGTTGGCAGGGTAACGGCCTACCAAGGCGACGATCAGTAGCCGGCCTGAGAGGGCGGACGGCCACACTGGGACTGAGACACGGCCCAGACTCCTACGGGAGGCAGCAGTTGGGGAATATTGCACAATGGGGGAAACCCTGATGCAGCGACGCCGCGTGAGTGAAGAAGTATTTCGGTATGTAAAGCTCTATCAGCAGGGAAGAAGATGACGGTACCTGACTAAGAAGCCCCGGCTAACTACGTGCCAGCAGCCGCGGTAATT

>LK5::ISJPSFI02G2EM4 5OTU578 454-44

TATAAGAGAGGGTTTGATCATGGCTCAGGACGAACGCTGGCGGCATGCCTAACACATGCAAGTCGAACGGAGTTATTATTCAGAAGTTCTTCGGGATGGAAGGATAAGAACTTAGTGGCGGACGGGTGAGTAACGCGTGGGTAACCTGCCCTTTTGTGGGGAACAACTTCGAGAAATCGGAGCTAATACCGCATAATAATAGAGGATCGCATGATTCTTTAAGGAAAGATGGCCTCTGAAGATGCTATCGCAAAAGGATGGACCCGCGTCTGATTAGCTAGTTGGAAGGGTAACGGCCTACCAAGGCAACGATCAGTAGCCGGCCTGAGAGGTTGAACGGCCACATTGGGACTGAGACACGGCCCAAACTCCTACGGGAGGCAGCAGTGGGGGATATTGCACAATGGGGGGAACCCTGATGCAGCGACGCCGCGTGAGTGAAGAAGCGTCTCGGCGCGTAAAGCTCTATCGGCAGGGAAGAGGGGGGCGACCCCGGACGGTACCTGACTAAGAAGCCCCGGCTAACTACGTGCCAGCAGCC

>LK5::ISJPSFI02H4KY4 5OTU584 454-45

TATAAGAGAGAGTTTGATCCTGGCTCAGGATGAACGCTGGCGGCGTGCCTAACACATGCAAGTCGAACGGGTGTACGGGATGGAAGATTTCGGTCGGAAGCCCCGTGCATGAGTGGCGGACGGGTGAGTAACGCGTGGGCAACCTGCCCTGTACAGGGGGACAACACTTAGAAATAGGTGCTAATACCGCATAACGGGAGAAGCCGCATGGCTTTTTCCTGAAAACTCCGGTGGTACAGGATGGGCCCGCGTCTGATTAGCCAGTTGGCAGGGTAACGGCCTACCAAAGCGACGATCAGTAGCCGGCCTGAGAGGGCGGACGGCCACACTGGGACTGAGACACGGCCCAGACTCCTACGGGAGGCAGCAGTGGGGGATATTGCACAATGGGGGGAACCCTGATGCAGCGACGCCGCGTGGGTGAAGAAGCGCCTCGGCGCGTAAAGCCCTGTCAGCAGGGAGAAAATGACGGTACCTGAAGAAGAAGCCCCGGCTAACTACGTGCCAGCAGCCGCGGTAAT

>LK6::ISJPSFI02F0OS8 6OTU034 454-47

TATAATGAAGAGTTTGATCATGGCTCAGGACGAACGCTGGCGGCGTGCCTAACACATGCAAGTCGAACGAAGCTTGATTCTTGATTTCTTCGGAAAGATAGATGATATGACTGAGTGGCGGACGGGTGAGTAACGCGTGAGCAACCTGCCCTTCGGAGGGGGATAGTGTCTGGAAACGGACAGTAATACCGCATAAAATATATTTGCCGCATGACAGATATATCAAAACTGAGGTGCCGAGGGATGGGCTCGCGTTGGATTAGATAGTTGGTGGGTAACGGCCTACCAAGTCAACGATCCATAGCCGGACTGAGAGGTTGAACGGCCACATTGGGACTGAGACACGGCCCAGACTCCTACGGGAGGCAGCAGTGGGGAATATTGCACAATGGGGGAAACCCTGATGCAGCAACGCCGCGTGAAGGAAGACGGTTTTTCGGATTGTAAACTTCTGTTCTTAGTGAAGAAGAATGACGGTAGCTAAGGAGCAAGCCACGGCTAACTACGTGCCAGCAGCC

>LK6::ISJPSFI02F9Q1D 6OTU049 454-48

TATAATGAAGGGTTTGATCCTGGCTCAGGATGAACGCTGGCGGCGTGCCTAACACATGCAAGTCGAGCGGAGCGCGGCGCTTCGAATTCTTCGGAAGGAAGAGCATCGCGTCTTAGCGGCGGACGGGTGAGTAACGCGTGGGCAACCTGCCTCATACAGGGGGATAACAGCCAGAAATGGCTGCTAATACCGCATAAGGCCACAGCACCGCATGGTGCAGCGGCAAAAAACTCCGGTGGTATGAGATGGGCCCGCGTCTGATTAGCTAGTTGGCAGGGTAACGGCCTACCAAGGCGACGATCAGTAGCCGGCCTGAGAGGGTGGACGGCCACATTGGGACTGAGACACGGCCCAAACTCCTACGGGAGGCAGCAGTGGGGGATATTGCACAATGGGGGAACCCTGATGCAGCGACGCCGCGTGAGCGATGAAGTATTTCGGTATGTAAAGCTCTATCAGCAGGGAGAAAATGACGGTACCTGACTAAGAAGCCCCGGCTAACTACGT

>LK6::ISJPSFI02F3ZZ2 6OTU065 454-49

TATAATGAAGGGTTTGATCATGGCTCAGGATGAACGCTGGCGGCGTGCTTAACACATGCAAGTCGAACGGAAATGATACGCTGATGCGATTTCGGTCAAATCATGTATCATTTTAGTGGCGGACGGGTGAGTAACGCGTGGGTAACCTGCCTTACACCGGGGGATAACACCTGGAAACAGGTGCTAATACCGCATAAGCGCACGGGAGCGCATGATCCTGTGTGAAAAACTCCGGTGGTGTAAGATGGACCCGCGTCTGATTAGCTTGTTGGCGGGGTAACGGCCCACCAAGGCGACGATCAGTAGCCGGCCTGAGAGGGTGGACGGCCACATTGGGACTGAGACACGGCCCAGACTCCTACGGGAGGCAGCAGTGGGGAATATTGCACAATGGGGGAAACCCTGATGCAGCGACGCCGCGTGAGCGAAGAAGTATTTCGGTATGTAAAGCTCTATCAGCAGGGAAGAAAAAATGACGGTACCTGAGTAAGAAGCACCGGCTAAATACGTGCCAGCAGCCGC

>LK6::ISJPSFI02G122S 6OTU073 454-50

TATAATGAAGGGTTTGATCATGGCTCAGGATGAACGCTGGCGGCGTGCCTAACACATGCAAGTCGAACGGAGTTACGATGAAACCTAGTGATTCGTAACTTAGTGGCGGACGGGTGAGTAACGCGTGGATAACCTGCCTTGCACTGGGGGATAACACTTAGAAATAGGTGCTAATACCGCATAAGCGCACAGAGCCGCATGGCTCAGTGTGAAAAACTCCGGTGGTGTAAGATGGATCCGCGTCTGATTAGCTGGTTGGCGGGGTAAAGGCCCACCAAAGCGACGATCGGTAGCCGGCCTGAGAGGGCGGACGGCCACATTGGGACTGAGACACGGCCCAGACTCCTACGGGAGGCAGCAGTGGGGGATATTGCACAATGGGGGGAACCCTGATGCAGCGACGCCGCGTGGGTGAAGGAGCGTTTCGGCGCGTAAAGCCCTATCGGCAGGGAAGAAGGAGGACGGTACCTGACTAAGAAGCCCCGGCTAACTACGTGCCAGCAGCCGCGGTAAT

>LK6::ISJPSFI02F0RZ0 6OTU083 454-51

TATAATGAAGAGTTTGATCCTGGCTCAGGATGAACGCTGGCGGCGTGCCTAACACATGCAAGTCGAGCGGAGCGCGGCGCTTCGAATTCTTCGGAAGGAAGGAGCATCGCGTCTTAGCGGCGGACGGGTGAGTAACGCGTGGGCAACCTGCCTCATACAGGGGGATAACAGCCAGAAATGGCTGCTAATACCGCATAAGGCCACAGCACCGCATGGTGCAGCGGCAAAAAACTCCGGTGGTATGAGATGGGCCCGCGTCTGATTAGCTAGTTGGCAGGGTAACGGCCTACCAAGGCGACGATCAGTAGCCGGCCTGAGAGGGCGGACGGCCACACTGGGACTGAGACACGGCCCAGACTCCTACGGGAGGCAGCAGTGGGGGATATTGCACAATGGGGGAACCCTGATGCAGCGACGCCGCGTGGGTGAAGGAGCGTTTCGGCGCGTAAAGCCCTATCGGCAGGGAAGAAGGAGGACGGTACCTGACTAAGAAGCCCCGGCTAACTACGTGCCAGCA

>LK6::ISJPSFI02F426R 6OTU084 454-52

TATAATGAAGAGTTTGATCCTGGCTCAGGATGAACGCTGGCGGCGTGCCTAACACATGCAAGTCGAACGGGTGTACGGGATGGAAGATTTCGGTCGGAAGCCCCGTGCATGAGTGGCGGACGGGTGAGTAACGCGTGGGCAACCTGCCCTGTACAGGGGGACAACACTTAGAAATAGGTGCTAATACCGCATAACGGGGGGAGCCGCATGGCACCCCCCTGAAAACTCCGGTGGTACAGGATGGGCCCGCGTCTGATTAGCTGGTTGGCAGGGTAACGGCCCACCAAGGCGACGATCAGTAGCCGGCCTGAGAGGGTGGACGGCCACATTGGGACTGAGACACGGCCCAAACTCCTACGGGAGGCAGCAGTGGGGGATATTGGACAATGGGGGAAACCCTGATCCAGCGACGCCGCGTGAGTGAAGAAGTATTTCGGTATGTAAAGCTCTGTCAGCAGGGAAGAAAGAAATGACGGTACCTGACCAAGAAGCCCCGGCTAACTACGTGCCAGCCGCCGCGGTAATT

>LK6::ISJPSFI02GGTIS 6OTU096 454-53

TATAATGAAGGGTTTGATCCTGGCTCAGGATGAACGCTGGCGGCGTGCCTAACACATGCAAGTCGAACGGGTGTACGGGATGGAAGATTTCGGTCGGAAGCCCCGTGCATGAGTGGCGGACGGGTGAGTAACGCGTGGGCAACCTGCCCTGTACAGGGGGACAACACTTAGAAATAGGTGCTAATACCGCATAACGGGGGGAGCCGCATGGCTTCCCCCTGAAAACTCCGGTGGTACAGGATGGGCCCGCGTCTGATTAGCTGGTTGGCAGGGTAACGGCCTACCAAGGCGACGATCAGTAGCCGGCCTGAGAGGGCGGACGGCCACATTGGGACTGAGACACGGCCCAGACTCCTACGGGAGGCAGCAGTGGGGAATATTGGACAATGGGGGAAACCCTGATGCAGCGACGCCGCGTGAGTGAAGAAGTATTTCGGTATGTAAAGCTCTATCAGCAGGGAAGAAGATGACGGTACCTGACTAAGAAGCCCCGGCTAACTACGTGCCAGCAGCCGCGGTAAT

>LK6::ISJPSFI02F5PAP 6OTU104 454-54

TATAATGAAGGGTTTGATCATGGCTCAGGACGAACGCTGGCGGCGTGCCTAACACATGCAAGTCGAACGAAGCTTGATTCTTGATTTCTTCGGAAAGATAGATGATATGACTGAGTGGCGGACGGGTGAGTAACGCGTGAGCAACCTGCCCGTTCGGAGGGGGATAGTGTCTGGAAACGGACAGTAATACCGCATAAAATATATTTGCCGCATGACAGATATATCAAAACTGAGGTGCCGAGGGATGGGCTCGCGTTGGATTAGATAGTTGGTGGGGTAACGGCCTACCAAGTCAACGATCCATAGCCGGACTGAGAGGTTGAACGGCCACATTGGGACTGAGACACGGCCCAAACTCCTACGGGAGGCAGCAGTGGGGAATATTGCACAATGGGGGAAACCCTGATGCAGCGACGCCGCGTGACGTGAAGAAGTATTTCGGTATGTAAAGCTCTATCAGCAGGGAAGAAGATGACGGTACCTGGACTAAGAAGCCCCGGCTAACTACGTGCCAGCAGCC

>LK6::ISJPSFI02GGTIS 6OTU105 454-55

TATAATGAAGGGTTTGATCCTGGCTCAGGATGAACGCTGGCGGCGTGCCTAACACATGCAAGTCGAACGGGTGTACGGGATGGAAGATTTCGGTCGGAAGCCCCGTGCATGAGTGGCGGACGGGTGAGTAACGCGTGGGCAACCTGCCCTGTACAGGGGGACAACACTTAGAAATAGGTGCTAATACCGCATAACGGGGGGAGCCGCATGGCTTCCCCCTGAAAACTCCGGTGGTACAGGATGGGCCCGCGTCTGATTAGCTGGTTGGCAGGGTAACGGCCTACCAAGGCGACGATCAGTAGCCGGCCTGAGAGGGCGGACGGCCACATTGGGACTGAGACACGGCCCAGACTCCTACGGGAGGCAGCAGTGGGGAATATTGGACAATGGGGGAAACCCTGATGCAGCGACGCCGCGTGAGTGAAGAAGTATTTCGGTATGTAAAGCTCTATCAGCAGGGAAGAAGATGACGGTACCTGACTAAGAAGCCCCGGCTAACTACGTGCCAGCAGCCGCGGTAAT

>LK6::ISJPSFI02F5MR6 6OTU118 454-56

TATAATGAAGAGTTTGATCCTGGCTCAGGATGAACGCTGGCGGCGTGCCTAACACATGCAAGTCGAGCGAAGCGCTTGAATACGATTCTTCGGATGAAGATTCTTGCGACTGAGCGGCGGACGGGTGAGTAACGCGTGGGCAACCTGCCTTACACAGGGGGATAACAGTTAGAAATGACTGCTAATACCGCATAAGACCACAAAGCTGCATGGCTAAGTGGTAAAAACTCCGGTGGTGTAAGATGGGCCCGCGTCTGATTAGGTAGTTGGTGGGGTAACGGCCCACCAAGCCGACGATCAGTAGCCGACCTGAGAGGGTGACCGGCCACATTGGGACTGAGACACGGCCCAGACTCCTACGGGAGGCAGCAGTGGGGAATATTGCACAATGGGGGAAACCCTGATGCAGCGACGCCGCGTGAGCGAAGAAGTATTTCGGTATGTAAAGCTCTATCAGCAGGGAGAAAATGACGGTACCTGAGTAAGAAGCTCCGGCTAAATACGTGCCAGCCGCCGCGGTAA

>LK6::ISJPSFI02GCIW1 6OTU198 454-57

TATAATGAAGGGTTTGATCATGGCTCGGGATGAACGCTGGCGGCGTGCTTAACACATGCAAGTCGAACGGAAATGATACGCTGATGCGATTTCGGTCAAATCATGTATCATTTTAGTGGCGGACGGGTGAGTAACGCGTGGGTAACCTGCCTTACACCGGGGGATAACACCTGGAAACAGGTGCTAATACCGCATAAGCGCACGGGAGCGCATGATCCTGTGTGAAAAACTCCGGTGGTGTAAGATGGACCCGCGTCTGATTAGCTTGTTGGCGGGGTAACGGCCCACCAAGGCGACGATCAGTAGCCGGCCTGAGAGGGTGGACGGCCACATTGGGACTGAGACACGGCCCAGACTCCTACGGGAGGCAGCAGTGGGGGATATTGCACAATGGGGGGAACCCTGATGCAGCGACGCCGCGTGAGTGAAGAAGCGCCTCGGCGCGTAAAGCTCTGTCAGCAGGGAAGAAGATGACGGTACCTGACCAAGAAGCCCCGGCTAACTACGTGCCAGCCGCCGCGGTAA

>LK6::ISJPSFI02FRG1U 6OTU201 454-58

TATAATGAAGGGTTTGATCCTGGCTCAGGATGAACGCTGGCGGCGTGCCTAACACATGCAAGTCGAACGGGTGTACGGGATGGAAGATTTCGGTCGGAAGCCCCGTGCATGAGTGGCGGACGGGTGAGTAACGCGTGGGCAACCTGCCCTGTACAGGGGGACAACACTTAGAAATAGGTGCTAATACCGCATAACGGGGGGAGCCGCATGGCTTCCCCCTGAAAACTCCGGTGGTACAGGATGGGCCCGCGTCTGATTAGCTGGTTGGCAGGGTAACGGCCTACCAAGGCGACGATCAGTAGCCGACCTGAGAGGGTGACCGGCCACATTGGGACTGAGACACGGCCCAGACTCCTACGGGAGGCAGCAGTGGGGAATATTGCACAATGGGGGAAACCCTGATGCAGCGACGCCGCGTGAGCGATGAAGTATTTCGGTATGTAAAGCTCTATCAGCAGGGAAGAAAATGACGGTACCTGACTAAGAAGCCCCGGCTAACTACGTGCCAGCAGCCGCGGTAATT

>LK6::ISJPSFI02F6857 6OTU312 454-59

TATAATGAAGAGTTTGATCCTGGCTCAGGACGAACGCTGGCGGCGTGCCTAACACATGCAAGTCGAACGAAGCTTGATTCTTGATTTCTTCGGAAAGATAGATGATATGACTGAGTGGCGGACGGGTGAGTAACGCGTGAGCAACCTGCCCTTCGGAGGGGGATAGTGTCTGGAAACGGACAGTAATACCGCATAAAATATATTTGCCGCATGACAGATATATCAAAACTGAGGTGCCGAGGGATGGGCTCGCGTTGGATTAGATAGTTGGTGGGGTAACGGCCTACCAAGTCAACGATCCATAGCCGGACTGAGAGGTTGAACGGCCACATTGGGACTGAGACACGGCCCAGACTCCTACGGGAGGCAGCAGTGGGGAATATTGCACAATGGGGGGAACCCTGATGCAGCGACGCCGCGTGGGTGAAGGAGCGTTTCGGCGCGTAAAGCCCTTTCGGCGGGGGAGAACGATGACGGTACCCGACGAAGAAGCACCGGCTAAATACGTGCCAGCCGCCGCGGTAATT

>LK6::ISJPSFI02F4KVP 6OTU331 454-60

TATAATGAAGGGTTTGATCCTGGCTCAGGATGAACGCTGGCGGCGTGCTTAACACATGCAAGTCGAACGGAAATGATACGCTGATGCGATTTCGGTCAAATCATGTATCATTTTAGTGGCGGACGGGTGAGTAACGCGTGGGTAACCTGCCTTACACCGGGGGATAACACCTGGAAACAGGTGCTAATACCGCATAAGCGCACGGGAGCGCATGATCCTGTGTGAAAAACTCCGGTGGTGTAAGATGGACCCGCGTCTGATTAGCTTGTTGGCGGGGTAACGGCCACCAAGGCGACGATCAGTAGCCGGCCTGAGAGGGTGGACGGCCACATTGGGACTGAGACACGGCCCAAACTCCTACGGGAGGCAGCAGTGGGGGATATTGGACAATGGGGGAAACCCTGATCCAGCGACGCCGCGTGAGTGAAGAAGTATTTCGGTATGTAAAGCTCTGTCAGCAGGGAAGAAAGAAATGACGGTACCTGACCAAGAAGCCCCGGCTAACTACGTGCCAGCCGCCGCGGTAATT

>LK6::ISJPSFI02FKSSR 6OTU350 454-61

TATAATGAAGGGTTTGATCATGGCTCAGGATGAACGCTGGCGGCGTGCCTAACACATGCAAGTCGAGCGGAGCGCGGCGCTTCGAATTCTTCGGAAGGAAGAGCATCGCGTCTTAGCGGCGGACGGGTGAGTAACGCGTGGGCAACCTGCCTCATACAGGGGGATAACAGCCAGAAATGGCTGCTAATACCGCATAAGGCCACAGCACCGCATGGTGCAGCGGCAAAAAACTCCGGTGGTATGAGATGGGCCCGCGTCTGATTAGCTAGTTGGCAGGGTAACGGCCTACCAAGGCGACGATCAGTAGCCGGCCTGAGAGGGTGGACGGCCACATTGGGACTGAGACACGGCCCAGACTCCTACGGGAGCAGCAGTGGGGGATATTGCACAATGGGGGAACCCTGATGCAGCGACGCCGCGTGGGTGAAGGAGTATTTCGGTATGTAAAGCTCTATCAGCAGGGAAGAAAATGACGGTACCTGACCAAGAAGCCCCGGCTAACTACGTGCCA

>LK6::ISJPSFI02G9S50 6OTU631 454-62

TATAATGAAGGGTTTGATCCTGGCTCAGGATGAACGCTGGCGGCGTGCCTAACACATGCAAGTCGAACGGGTGTACGGGATGGAAGATTTCGGTCGGAAGCCCCGTGCATGAGTGGCGGACGGGTGAGTAACGCGTGGGCAACCTGCCCTGTACAGGGGGACAACACTTAGAAATAGGTGCTAATACCGCATAACGGGGGGAGCCGCATGGCTTCCCCCTGAAAACTCCGGTGGTACAGGATGGGCCCGCGTCTGATTAGCTGGTTGGCAGGGTAACGGCCTACCAAGGCGACGATCAGTAGCCGGCCTGAGAGGGCGGACGGCCACACTGGGACTGAGACACGGCCCAGACTCCTACGGGAGGCAGCAGTGGGGGATATTGCACAATGGGGGAAACCCTGATGCAGCGACGCCGCGTGGGTGAAGAAGTACTTCGGTACGTAAAGCCCTATCAGCAGGGAAGAAAATGACGGTACCTGATTAAGAAGCCCCGGCTAACTACGTGCCAGCAGCCGCGGTAAT

>LK7::ISJPSFI02F2GWE 7OTU040 454-63

TATAATGGAGAGTTTGATCCTGGCTCAGGACGAACGCTGGCGGCGTGCCTAACACATGCAAGTCGAACGAAGCTTGACGAATGATTTCTTCGGAATGAAATCTGATATGACTGAGTGGCGGACGGGTGAGTAACGCGTGAGCAACCTGCCCTTCGGAGGGGGATAGTGTCTGGAAACGGACAGTAATACCGCATAACGTATTTTTACCGCATGATAGAAATACCAAAACTGAGGTGCCGAAGGATGGGCTCGCGTTGGATTAGATAGTTGGTGGGGTAACGGCCTACCAAGTCGACGATCCATAGCCGGCCTGAGAGGGCGGCCGGCCACATTGGGACTGAGACACGGCCCAAACTCCTGCGGGAGGCAGCAGTGGGGAATATTGCACAATGGGGGAAACCCTGATGCAGCAACGCCGCGTGAGTGAAGAAGTATTTCGGTACGTAAAGCTCTATCAGCAGGAAAGAAAATGACGGTACCTGACTAAGAAGCCCCGGCTAACTACGTGCCAGCAGCCGCGGTAAT

>LK7::ISJPSFI02F0TSP 7OTU045 454-64

TATAATGGAGAGTTTGATCCTGGCTCAGAACGAACGCTGGCGGCGTGGATAAGACATGCAAGTCGAACGAGAGAATTGCTAGCTTGCTAATAATTCTCTAGTGGCGCACGGGTGAGTAACACGTGAGTAACCTGCCTCCGAGAGTGGGATAGCCCTGGGAAACTGGGATTAATACCGCATAGAATCGCAAGATTAAAGCAGCAATGCGCTTGGGGATGGGCTCGCGGCCTATTAGTTAGTTGGTGAGGTAACGGCTCACCAAGGCGATGACGGGTAGCCGGTCTGAGAGGATGTCCGGCCACACTGGAACTGAGACACGGTCCAGACACCTACGGGTGGCAGCAGTCGAGAATCATTCACAATGGGGGAAACCCTGATGGTGCGACGCCGCGTGGGGGAATGAAGGTCTTCGGATTGTAAACCCCTGTCATGTGGGAGCAAATTAAAAGATAGTACCACAAGAGGAAGAGACGGCTAACTCTGTGCCAGCAGCCGCGGTAATT

>LK7::ISJPSFI02FPK7H 7OTU057 454-65

TATAATGGAGGGTTTGATCATGGCTCAGGATGAACGCTGGCGGCGTGCCTAACACATGCAAGTCGAACGGGTGTACGGGGAGGAAGGCTTCGGCCGGAAAACCTGTGCATGAGTGGCGGACGGGTGAGTAACGCGTGGGCAACCTGGCCTGTACAGGGGGATAACACTTAGAAATAGGTGCTAATACCGCATAACGGGGGAAGCCGCATGGCTTTTCCCTGAAAACTCCGGTGGTACAGGATGGGCCCGCGTCTGATTAGCCAGTTGGCAGGGTAACGGCCTACCAAAGCGACGATCAGTAGCCGGCCTGAGAGGGCGGACGGCCACACTGGGACTGAGACACGGCCCAGACTCCTACGGGAGGCAGCAGTGGGGAATATTGGGCAATGGGCGCAAGCCTGACCCAGCAACGCCGCG

>LK7::ISJPSFI02F09NU 7OTU059 454-66

TATAATGGAGGGTTTGATCATGGCTCAGGATGAACGCTGGCGGCGTGCCTAACACATGCAAGTCGAACGAAGCTTGACGAATGATTTCTTCGGAATGAAATCTGATATGACTGAGTGGCGGACGGGTGAGTAACGCGTGAGCAACCTGCCCTTCGGAGGGGGATAGTGTCTGGAAACGGACAGTAATACCGCATAACGTATTTTTACCGCATGATAGAAATACCAAAACTGAGGTGCCGAAGGATGGGCTCGCGTTGGATTAGATAGTTGGTGGGGTAACGGCCCACCAAGGCAGCGATCAGTAGCCGGCCTGAGAGGGTGGACGGCCACATTGGGACTGAGACACGGCCCAGACTCCTACGGGAGGCAGCAGTGGGGAATATTGGGCAATGGGCGCAAGCCTGACCCAGCAACGCCGCGTGAAGGAAGAAGGCTTTCGGGTTGTAAACTTCTTTTCTGAGGGACGAAGAAAGTGACGGTACCTCAGGAATAAGCCACGGCTAACTACGTGCCAGCAGCCGCGGTAATTGAAGGAAGAAGGCTTTCGGGTTGTAAACTTCTTTTCTCAGGGACGAAGCAAGTGACGGTACCTGAGGAATAAGCCACGGCTAACTACGTGCCAGCAGCCGCGGTAATT

>LK7::ISJPSFI02F9RPB 7OTU066 454-67

TATAATGGAGGGTTTGATCATGGCTCAGGACGAACGCTGGCGGCGTGCTTAACACATGCAAGTCGAACGGAGTACCCTCGAAAGAGATTTCGGTCAATGGAGAGGACTACTTAGTGGCGGACGGGTGAGTAACGCGTGAGGAACCTGCCTTTCAGTGGGGGACAACAGTTGGAAACGACTGCTAATACCGCATAACGTACTGGTATCGCATGGTACTGGTACCAAAGATTTATCGCTGAGAGATGGCCTCGCGTCTGATTAGCTAGTTGGTAGGGTAACGGCCTACCAAGGCGACGATCAGTAGCCGGACTGAGAGGTTGGCCGGCCACATTGGGACTGAGATACGGCCCAGATTCCTACGGGAGGCAGCAGTGGGGAATATTGGGCAATGGGCGCAAGCCTGACCCAGCAACGCCGCGTGAAGGAAGAAGGCTTTCGGGTTGTAAACTTCTTTTGTCAGGGACGAAGCAAGTGACGGTACCTGACGAATAAGCCACGGCTAACTACGTGCCAGCAGCCGCGGTAATT

>LK7::ISJPSFI02GAG37 7OTU116 454-68

TATAATGGAGGGTTTGATCATGGCTCAGGACGAACGCTGGCGGCGTGCCTAACACATGCAAGTCGAACGAAGCTTGACGAATGATTTCTTCGGAATGAAATCTGATATGACTGAGTGGCGGACGGGTGAGTAACGCGTGAGCAACCTGCCCTTCGGAGGGGGATAGTGTCTGGAAACGGACAGTAATACCGCATAACGTATTTTTACCGCATGATAGAAATACCAAAACTGAGGTGCCGAAGGATGGGCTCGCGTTGGATTAGATAGTTGGTGGGGTAACGGCCTACCAAGTCGACGATCAGTAGCCGGCCTGAGAGGGCGGACGGCCACACTGGGACTGAGACACGGCCCAGACTCCTACGGGAGGCAGCAGTGGGGGATATTGCACAATGGGGGGAACCCTGATGCAGCGACGCCGCGTGGGTGAAGAAGCGCCTCGGCGCGTAAAGCCCTGTCAGCAGGGAAGAAAATGACGGTACCTGAAGAAGAAGCCCCGGCTAACTACGTGCCAGCAGCCGCGGTAAT

>LK8::ISJPSFI02F4SU7 8OTU014 454-69

TATATCTCAGGGTTTGATCATGGCTCAGGATGAACGCTGGCGGCATGCCTAATACATGCAAGTCGAACGAGAGACCTTCGGGTCTCTAGTGGCGAACGGGTGAGTAACACGTAGGGAACCTGCCCGCGCACCGGGAATACGCTCTGGAAACGGAGAACAAATCCCGATGTACAGGAAGGAGGCATCTTCTTTCTGTGAAACATCCTTTAGGGGATGGGGCGCGGATGGACCTGCGGTGCATTAGTTTGTTGGCGGGGTAAAGGCCCACCAAGACGATGATGCATAGCCGGCCTGAGAGGGCGGACGGCCACATTGGGACTGAGACACGGCCCAGACTCCTGCGGGAGGCAGCAGTAGGGAATTTTCGTCAATGGGCGCAAGCCTGAACGAGCGATGCCGCGTGAGTGAAGAAGGCCTTCGGGTCGTAAAGCTCTGTTGCGGGGGAAAAAAGGCAGCATCAGGAAATGGGTGCTGACTGATGGTGCCCCGCCAGAAAGTCACGGCTAACTACGTGCCAGCA

>LK8::ISJPSFI02F2D0F 8OTU020 454-70

TATATCTCAGAGTTTGATCATGGCTCAGGATGAACGCTGGCGGCGTGCTTAACACATGCAAGTCGAACGGAAATGATACGCTGAAGCGATTTCGGTCAAATCATGTATCATTTTAGTGGCGGACGGGTGAGTAACGCGTGGGTAACCTGCCTTGCACTGGGGGATAACACTTAGAAATAGGTGCTAATACCGCATAAGCGCACGAGACCGCATGGTCTAGTGTGAAAAACTCCGGTGGTGTAAGATGGACCCGCGTCTGATTAGCTAGTTGGCGGGTAACGGCCTACCAAGGCGACGATCAGTAGCCGGCCTGAGAGGGTGGACGGCCACATTGGGACTGAGACACGGCCCAAACTCCTACGGGAGGCAGCAGTGGGGGATATTGGACAATGGGGGAAACCCTGATCCAGCGACGCCGCGTGAGTGAAGAAGTATTTCGGTATGTAAAGCTCTATCAGCAGGGAAGAAAATGACGGTACCTGACTAAGAAGCCCCGG

>LK8::ISJPSFI02F8VKT 8OTU070 454-71

TATATCTCAGAGTTTGATCATGGCTCAGGATGAACGCTGGCGGCGTGCTTAACACATGCAAGTCGAACGAAGCGCGCGGAAGGAAGGCCTTCGGGCCGGAGAGCCGCGGGACTGAGTGGCGGACGGGTGAGTAACGCGTGGGCAACCTGCCCCGTACAGGGGGATAACAGCCGGAAACGGCTGCTAATACCGCATAAGCGCACAGCGCCGCATGGCGCGGTGTGAAAAGCTCCGGCGGTACGGGAGGGGCCCGCGTCTGATTAGGCAGTTGGCGGGGCAGCGGCCCACCAAACCGACGATCAGTAGCCGGCCTGAGAGGGCGGCCGGCCACATTGGGACTGAGACACGGCCCAAACTCCTACGGGAGGCAGCAGTGGGGAATATTGCACAATGGGGGAAACCCTGATGCAGCGACGCCGCGTGAAGGAAGAAGTATTTCGGTATGTAAACTTCTATCAGCAGGGAAGAAGATGACGGTACCTGACTAAGAAGCCCCGGCTAACTACGTGCCAGCAGCCGCGGTAAT

>LK8::ISJPSFI02G1USB 8OTU092 454-72

TATATCTCAGAGTTTGATCATGGCTCAGGATGAACGCTGGCGGCGTGCTTAACACATGCAAGTCGAACGGAAATGATACGCTGAAGCGATTTCGGTCAAATCATGTATCATTTTAGTGGCGGACGGGTGAGTAACGCGTGGGTAACCTGCCTTGCACTGGGGGATAACACTTAGAAATAGGTGCTAATACCGCATAAGCGCACGAGACCGCATGGTCTAGTGTGAAAAACTCCGGTGGTGTAAGATGGACCCGCGTCTGATTAGCTAGTTGGCGGGGTAACGGCCTACCAAGGCGACGATCAGTAGCCGGCCTGAGAGGGTGGACGGCCACATTGGGACTGAGACACGGCCCAAACTCCTACGGGAGGCAGCAGTGGGGGATATTGGACAATGGGGGGAACCCTGATGCAGCGACGCCGCGTGGGTGAAGAAGCGCCTCGGCGCGTAAAGCCCTGTCAGCAGGGAAGAAAATGACGGTACCTGAAGAAGAAGCCCCGGCTAACTACGTG

>LK8::ISJPSFI02F0VD6 8OTU115 454-73

TATATCTCAGGGTTTGATCCTGGCTCAGGATGAACGCTGGCGGCGTGCTTAACACATGCAAGTCGAACGGAAATGATGCGCTGATGCGATTTCGGTCAAATCTTGTATCATTTTAGTGGCGGACGGGTGAGTAACGCGTGGGTAACCTGCCTTGCACTGGGGGATAACACTTAGAAATAGGTGCTAATACCGCATAACGGGAGAAGCCGCATGGCTTTTTCCTGAAAACTCCCGTGGTACAGGATGGGCCCGCGTCTGATTAGCCAGTTGGCAGGGTAACGGCCTACCAAAGCGACGATCAGTAGCCGGCCTGAGAGGGCGGACGGCCACACTGGGACTGAGACACGGCCCAGACTCCTACGGGAGGCAGCAGTGGGGGATATTGCACAATGGGGGGAACCCTGATGCAGCGACGCCGCGTGGGTGAAGAAGCGCCTCGGCGCGTAAAGCCCTGTCAGCAGGGAGAAAATGACGGTACCTGAAGAAGAAGCCCCGGCTAACTACGTGCCAGCAGCCGCGGTAA

>LK8::ISJPSFI02FT3S7 8OTU243 454-74

TATATCTCAGGGTTTGATCATGGCTCAGGATGAACGCTGGCGGCGTGCTTAACACATGCAAGTCGAACGGAAATGATGCGCTGATGCGATTTCGGTCAAATCTTGTATCATTTTTAGTGGCGGACGGGTGAGTAACGCGTGGGTAACCTGCCTTGCACTGGGGGATAACACCTAGAAATAGGTGCTAATACCGCATAAGCGCACGAGACCGCATGGTTTTGTGTGAAAAACTCCGGTGGTGTAAGATGGACCCGCGTCTGATTAGCTAGTTGGCGGGGTAACGGCCCACCAAGGCGACGATCAGTAGCCGGCCTGAGAGGGTGGACGGCCACATTGGGACTGAGACACGGCCCAAACTCCTACGGGAGGCAGCAGTGGGGGATATTGCACAATGGGGGAAACCCTGATGCAGCGACGCCGCGTGAGCGAGGAAGTATTTCGGTATGTAAAGCTCTGTCAGCAGGGAAGAAAGATGACGGTACCTGACCAAGAAGCACCGGCTAAATACGTGCCAGCAGCC

>LK8::ISJPSFI02G0O2C 8OTU281 454-75

TATATCTCAGAGTTTGATCCTGGCTCAGGATGAACGCTGGCGGCGTGCCTAACACATGCAAGTCGAACGGGTGCGCGCAGGAAGGGCGGGGTCAAACCGGTCCCGGAGGGTGCGCATTGAGTGGCGGACGGGTGAGTAACGCGTGGGCAACCTGCCGTATACAGGGGGATAACACCCGGAACGGGTGCTAATACCGCATAAGCGCACGAGTGCCGCATGGCACGGTGTGAAAAACTCCGGTGGTATACGATGGGCCCGCGTCCGATTAGCTTGTTGGCGGGGCAGCGGCCCACCAAGGCGACGATCAGTAGCCGGGCCTGAGAGGGTGGACGGCCACATTGGGACTGAGACACGGCCAAACTCCTACGGGAGGCAGCAGTGGGGATATTGGACAATGGGGGAAACCCTGATCCAGCGACGCCGCGTGAGTGAAGAAGTATTTCGGTATGTAAAGCTCTGTCAGCAGGGAAGAAAGAAATGACGGTACCTGACCAAGAAGCCCCGGCTAACTACGTGCCAGCCGCCGCGGTAAT

>LK8::ISJPSFI02F8WR7 8OTU683 454-76

TATATCTCAGAGTTTGATCCTGGCTCAGGATGAACGCTGGCGGCGTGCCTAACACATGCAAGTCGAACGGGTGTACGGGGAGGAAGGCTTCGGCCGGAAAACCTGTGCATGAGTGGCGGACGGGTGAGTAACGCGTGGGCAACCTGGCCTGTACAGGGGGATAACACTTAGAAATAGGTGCTAATACCGCATAACGGGGGAAGCCGCATGGCTTTTTCCTGAAAACTCCGGTGGTACAGGATGGGCCCGCGTCTGATTAGCCAGTTGGCAGGGTAACGGCCTACCAAGGCGACGATCAGTAGCCGGCCTGAGAGGGTGGACGGCCACATTGGGACTGAGACACGGCCCAAACTCCTACGGGAGGCAGCAGTGGGGGATATTGGACAATGGGGGAAACCCTGATCCAGCGACGCCGCGTGAGTGAAGAAGTATTTCGGTATGTAAAGCTCTGTCAGCAGGGAAGAAAGAAATGACGGTACCTGACCAAGAAGCCCCGGCTAACTACGTGCCAGCCGCCGCGGTAAT
